# Effectiveness of Gamification Interventions to Improve Physical Activity and Sedentary Behavior in Children and Adolescents: Systematic Review and Meta-Analysis

**DOI:** 10.2196/68151

**Published:** 2025-09-18

**Authors:** Min Wang, Jisheng Xu, Xulin Zhou, Xingchen Li, Yu Zheng

**Affiliations:** 1 School of Economics and Management, Chengdu Sport University, 1942 Huanhu North Road, Eastern New Area, Chengdu, 641418, China, 86 18980061501; 2 School of Sports Medicine and Health, Chengdu Sport University, Chengdu, China; 3 School of Physical Education, Sichuan Normal University, Chengdu, China

**Keywords:** gamification, physical activity, behavior change, eHealth, health behavior, intervention, mobile phone, meta-analysis, systematic review, PRISMA

## Abstract

**Background:**

Physical activity (PA) is critically linked to the health outcomes of children and adolescents. Gamification interventions represent a promising approach to promote PA engagement. However, the effects of these interventions on both PA and sedentary behavior (SB) in this population remain controversial. This review seeks to clarify this controversy.

**Objective:**

This systematic review aimed to evaluate the effectiveness of gamification interventions in enhancing PA and reducing SB in children and adolescents, while identifying potential moderators for PA promotion.

**Methods:**

We systematically searched PubMed, Web of Science, Embase, EBSCO, and Cochrane Library databases for randomized controlled trials (RCTs) published between January 1, 2010, and August 1, 2024. Included RCTs examined gamification interventions targeting PA, SB, daily step counts, and BMI in children and adolescents. Random-effects meta-analyses were performed using RevMan 5.4 (Cochrane) and Stata 18.0 (StataCorp), with subgroup analyses assessing moderating effects of theoretical paradigms, game elements, and intervention duration. Methodological robustness was evaluated via the Egger regression test, sensitivity analyses (leave-one-out method), and funnel plot inspection for publication bias.

**Results:**

A total of 16 RCTs involving 7472 children and adolescents (age range 6‐18 y) were included. Our findings showed that the gamification interventions significantly increased moderate-to-vigorous physical activity (MVPA; standardized mean difference [SMD] 0.15, 95% CI 0.01 to 0.29; *P*=.04) and reduced BMI (SMD 0.11, 95% CI 0.05 to 0.18; *P*<.001). However, there was no significant improvement in SB (SMD 0.07, 95% CI −0.07 to 0.22; *P*=.33), vigorous physical activity (SMD 0.12, 95% CI -0.3 to 0.55; *P*=.56), moderate physical activity (SMD 0.16, 95% CI −0.2 to 0.53; *P*=.38), light physical activity (SMD −0.00, 95% CI −0.49 to 0.48; *P*>.99), and daily step count (SMD 0.22, 95% CI −0.51 to 0.94; *P*=.55). Subgroup analyses revealed significant moderation effects for MVPA improvement by theoretical paradigm, game elements, intervention duration, and study setting.

**Conclusions:**

This meta-analysis confirms that gamification interventions effectively increased MVPA in children and adolescents, with sustained effects persisting beyond follow-up. The efficacy of these interventions is significantly moderated by theoretical paradigms, game elements, and intervention duration. However, blinding infeasibility contributed to prevalent performance bias, potentially introducing detection bias for subjective SB and PA metrics. Future research should strengthen blinding protocols for outcome assessors, enhance allocation concealment reporting, and validate conclusions through high-quality RCTs.

## Introduction

### Background

Regular physical activity (PA) is associated with numerous health benefits. However, most children and adolescents fail to meet global physical activity guidelines [1-3]. The World Health Organization (WHO) recommends that children and adolescents accumulate ≥60 minutes of daily moderate-to-vigorous physical activity (MVPA) [4]. Yet global surveillance indicates only 19.3% of 11‐17-year-olds meet this target, with average MVPA levels declining to 43.7 minutes/day in high-income countries [5]. Approximately 324 million adolescents (18% of the global adolescent population) live with overweight or obesity worldwide. Furthermore, insufficient PA contributes to rising rates of poor health outcomes and chronic diseases [6-9]. Conversely, regular PA can avert the risk of chronic diseases [10,11] and reduce the development of noncommunicable risk factors [12,13]. Epidemiological studies demonstrate a “dose-response” relationship between sedentary behavior (SB) and mortality [14]. High-level PA has been shown to attenuate or even eliminate the deleterious effects of SB on early adolescent mortality [14]. In this context, developing interventions to enhance PA and reduce SB represents a critical public health imperative. Given this pervasive deficit, gamification emerges as a targeted strategy to bridge the gap between adolescents’ digital behaviors and health needs.

Gamification based on electronic devices and immersive technologies is considered a promising intervention for changing health behaviors [15,16]. Leveraging adolescents’ fondness for digital products (with 95% of individuals in this age group using smartphones daily [17]), gamification strategies aim to transform PA into a goal-driven and rewarding experience. Defined as “the application of game-design elements in non-game contexts” [16], this approach incorporates gamification elements (eg, rewards, feedback, and social interactions) to create game-inspired experiences that enhance PA engagement [16].

However, current research predominantly focuses on adults and chronic conditions (eg, rheumatic diseases and cancer survivors) [18-22], where efficacy is established. In contrast, gamification’s impact on children and adolescents remains understudied. While existing literature examines gamification’s impact on PA or SB in healthy adolescents, intervention outcomes remain inconsistent [23,24]. Direito et al [25] suggest that underlying factors may influence gamification interventions for PA, including individual characteristics, environmental facilitators, and implementation fidelity. Although youth studies show limited total physical activity (TPA) or MVPA effects [26-28], these findings likely reflect methodological constraints rather than inherent limitations of PA interventions. Systematic reviews emphasize scarce high-quality studies and the need for rigorous randomized controlled trials (RCTs) to isolate gamification-specific effects [23,24,29]. Paradoxically, when using stringent experimental controls, gamification’s impact may attenuate [24]. Consequently, evidence regarding gamification’s effectiveness in promoting PA and reducing SB remains inconclusive, highlighting the need for meta-analyses of high-quality RCTs. Furthermore, gamification interventions based on theoretical paradigms may be more conducive to increasing PA in children and adolescents [30]. Several theoretical paradigms have been used for gamification interventions [31], including self-determination theory (SDT), transtheoretical models (TTMs), behavior change techniques (BCTs), and social cognitive theory (SCT). Different factors, such as game elements and theoretical paradigms, are crucial in determining the effectiveness of gamification interventions. Therefore, systematic evaluation is essential.

### This Study

This systematic review aims to assess the effectiveness of gamification interventions in promoting PA and reducing SB in children and adolescents. Specifically, (1) the aim is to evaluate the efficacy of a gamification intervention in improving MVPA, vigorous physical activity (VPA), moderate physical activity (MPA), light physical activity (LPA), daily step count, BMI, and reducing SB in children and adolescents. (2) The aim is to examine the effectiveness of gamification interventions across diverse subgroups, considering the moderating effects of variables, such as theoretical framework, game elements, and intervention cycle. In addition, unlike existing reviews, this review will assess the effectiveness of gamification interventions in promoting PA and reducing SB in children and adolescents based on rigorous research components derived from RCTs. Subgroup analyses will examine the influence of crucial moderating variables, including theoretical framework, game elements, and intervention duration, on the effectiveness of gamification interventions. This approach will improve our understanding of the factors that influence the effectiveness of these interventions and facilitate the accurate quantification of intervention programs.

## Methods

### Registration

This study was conducted in accordance with the PRISMA (Preferred Reporting Items for Systematic Reviews and Meta-Analyses) guidelines [32,33]. The detailed PRISMA checklist is provided in Checklist 1. The systematic review was registered on PROSPERO (International Prospective Register of Systematic Reviews; CRD42024580995).

### Search Strategy and Information Sources

MW and XL conducted independent literature searches according to the PRISMA 2020 statement. A comprehensive search of multiple databases was performed, including PubMed, Web of Science, Ebsco, Embase, and the Cochrane Library. The search period was limited to January 1, 2010 (2010 being the date of the widespread adoption of the term “gamification” [34]), and August 1, 2024. A literature search strategy was developed by 2 investigators using Boolean operators (“AND” and “OR”) to combine subject terms with open-access terms. The search terms used were “child” or “adolescent,” “gamification,” “exergaming,” “exert-gaming,” “gamified,” “gameful,” and “physical activity,” “PA,” “MVPA,” and “sedentary behavior.” The search terms “exergaming,” “exergaming,” “gamified,” and “graceful” were combined with the search terms “physical activity,” “PA,” “MVPA,” “sedentary behavior,” “SB,” and “sedentary behavior.” In addition, the search terms “sedentary lifestyle,” “sedentary behavior,” and “sedentary behavior” were included. A detailed description of the PubMed search strategy can be found in Multimedia Appendix 1. In addition, we supplemented our search results with references cited in previous reviews.

### Inclusion and Exclusion Criteria

#### Inclusion Criteria

The PICOS (Population, Intervention, Comparison, Outcome, and Study Design) principles developed the screening criteria, and the inclusion criteria for this study were as follows: (1) The study comprised children and adolescents, aged 6 to 18 years, with no gender or health status restrictions. Participants with chronic conditions (eg, obesity, diabetes) were included if their condition permitted safe physical activity participation under medical guidance. Individuals were excluded only if they had physical impairments contraindicating PA (eg, uncontrolled cardiomyopathy), had intellectual or cognitive disabilities affecting intervention adherence, and if they had medical conditions precluding safe exercise (eg, acute fractures). (2) Gamification interventions systematically apply game design elements (eg, points, leaderboards, and badges) within nongame contexts to enhance PA participation or reduce SB in pediatric populations. This approach explicitly differs from serious games (eg, Pokémon Go)—defined as purpose-built digital experiences with nonentertainment objectives such as health education or skill training [35]. Whereas serious games create immersive online environments, gamification leverages motivational mechanics embedded in existing activities, exemplified by wearable-tracked PA metrics synced with mobile apps to trigger reward systems [36]. To resolve definitional ambiguities highlighted in the literature, 3 researchers (MW, JX, and XL) established consensus criteria through the Delphi methodology. (3) Including regular exercise methods, a control group, a no-intervention group, and gamified interventions. (4) Primary outcome measures included MVPA, VPA, MPA, LPA, BMI, SB, and daily steps. These outcomes were obtained through objective measurements using accelerometers, pedometers, or smartphone apps. Due to the increased risk of data bias associated with subjective measures obtained via self-report questionnaires, objective data are consistently preferred over self-report questionnaires in analyses [37]. Consequently, outcome measures derived from self-report questionnaires were excluded from this study. (5) The study design was an RCT.

#### Exclusion Criteria

The exclusion criteria were as follows: (1) unavailability of original articles, review studies, conference abstracts, repetitive publications, animal experiments, and nonrandomized controls; (2) incomplete data and fruitless attempts to contact the authors of the articles; (3) non–English-language literature; and (4) literature in which the outcome metrics were not related to PA and SB. Textbox 1 shows the summary of the inclusion and exclusion criteria.[Table T1]

Textbox 1. **Summary of the inclusion and exclusion criteria.**

**Inclusion criteria:**

- Participants: Children and adolescents (ages 1-18 y), regardless of gender or health status, including those with chronic conditions, overweight, obesity, or other health issues not preventing participation.
- Intervention: Gamification-based interventions incorporating at least one game design element (eg, points, leaderboards, and badges), delivered through wearable devices, mobile apps, or related platforms.
- Comparator: Usual care, no intervention, waitlist controls, or active nongamified interventions (eg, standard exercise programs).
- Outcomes: Objectively measured MVPA, VPA, MPA, LPA, SB, BMI, and daily steps (eg, measured with accelerometers such as ActiGraph GT3X+, pedometers, or smartphone apps).
- Study design: Full-text English RCTs or cluster RCTs (2010‐2024).
- Data quality: Complete datasets.

**Exclusion criteria:**

- Participants: Adults, individuals with physical impairments or medical contraindications to physical activity (eg, uncontrolled cardiomyopathy or acute fractures), and children or adolescents with intellectual disabilities or cognitive impairments that hinder adherence to interventions.
- Intervention: Nongamified digital interventions or purely entertainment-focused games without systematic gamification elements.
- Comparator: Gamified control groups, noncomparative designs.
- Outcomes: Studies reporting only self-reported PA or SB outcomes, or those in which outcomes were unrelated to PA and SB.
- Study design: Nonrandomized trials, conference abstracts, review studies, repetitive publications, animal studies, and non-English literature.
- Data quality: Studies with incomplete datasets where attempts to contact authors were unsuccessful.

### Study Selection

The article screening process uses the PRISMA 2020 statement flowchart paradigm to document the studies selected or excluded at each step [33]. The search results were summarized and imported into EndNote 21 literature management software (Clarivate Analytics, Inc), where MW performed the initial screening by sorting and removing duplicates. To ensure the inclusion of original studies that met the established criteria, authors XL and JX independently assessed the title and abstract of the articles using EndNote 21 software. In addition, 2 researchers subjected all articles potentially meeting the criteria to a full-text review. Any discrepancies arising during the review process will be discussed and resolved with MW.

### Data Extraction

Furthermore, 2 authors (XZ and XL) used a standardized data abstraction form to extract the data. Any discrepancies were discussed and resolved with the second author (MW and YZ). The extracted content included the following elements: (1) Basic information (authors, publication year, country, and study design); (2) Population characteristics (sample size and age distribution); (3) Interventions: Gamification elements (points, leaderboards, and badges); (4) Intervention duration (weeks); (5) Control group protocols; (6) Outcome metrics (mean [SD] for MVPA, VPA, MPA, LPA, SB, step counts, and BMI); (7) Theoretical frameworks and measurement instruments (eg, ActiGraph model specifications).

### Risk of Bias Assessment

For each eligible trial, 2 reviewers (MW and XZ) used the specially constructed Cochrane Risk of Bias tool (outlined in the Cochrane Handbook for Evaluation of Intervention Systems [38]) to assess the risk of bias. The risk of bias was assessed in 7 domains: sequence generation, allocation concealment, blinding of subjects and personnel, blinding of outcome assessment, incomplete outcome data, selective outcome reporting, and other risks of bias. In this meta-analysis, we comprehensively assessed the potential risk of bias based on the content of the included literature. We categorized the studies as high risk, low risk, or unclear (if relevant information was unavailable). The results of these risk-of-bias assessments were documented in the review and included in the final analysis. To assess interrater agreement, we calculated the κ statistic. After a comprehensive assessment, we determined that the included studies were strikingly similar in design and that blinding was not feasible for this intervention. Consequently, we rated the risk of blinding bias for these studies as high. Given these considerations, the increased risk of bias associated with blinded programs is particularly evident in the risk of bias assessment plot.

### Statistical Analysis

A meta-analysis was performed using RevMan 5.4 software (Cochrane) and Stata17 software (StataCorp). This included calculating effect size combinations, performing heterogeneity tests, generating forest plots, performing subgroup analyses, assessing publication bias (using funnel plots and the Egger test [39]), and performing sensitivity analyses. The original study data set included continuous variables. Therefore, the standardized mean difference (SMD) and its 95% CI were selected as the effect measures for the pooled analysis. In addition, PA and SB data, including means and SDs, were extracted independently from each study. For some of the studies that did not provide these data directly, we supplemented them using the estimation method of Hozo et al [39]. The heterogeneity test of this study was performed using *I*^2^ and *P* values. When *I*² was less than 50%, and *P* was equal to or greater than .10, the data were analyzed using a fixed-effects model. Conversely, a meta-analysis used a random-effects model when *I*² exceeded 50%, and *P* was less than .10 [40]. Subgroup analyses will be performed to gain a deeper understanding of the impact of other potential factors on the outcome indicators. The potential for publication bias in the literature was assessed using funnel plots and the Egger test. Publication bias was inferred if the funnel plots showed asymmetry or if *P*<.05 in the Egger test. Otherwise, the possibility of publication bias was considered low. The effect size interpretation criteria were SMD<0.2 for negligible effect sizes, 0.2≤SMD<0.5 for small effect sizes, 0.5≤SMD<0.8 for moderate effect sizes, and SMD≥0.8 for large effect sizes. A Z statistic of *P*<.05 was used to assess the significance of the overall effect.

## Results

### Study Selection

**Figure 1.**
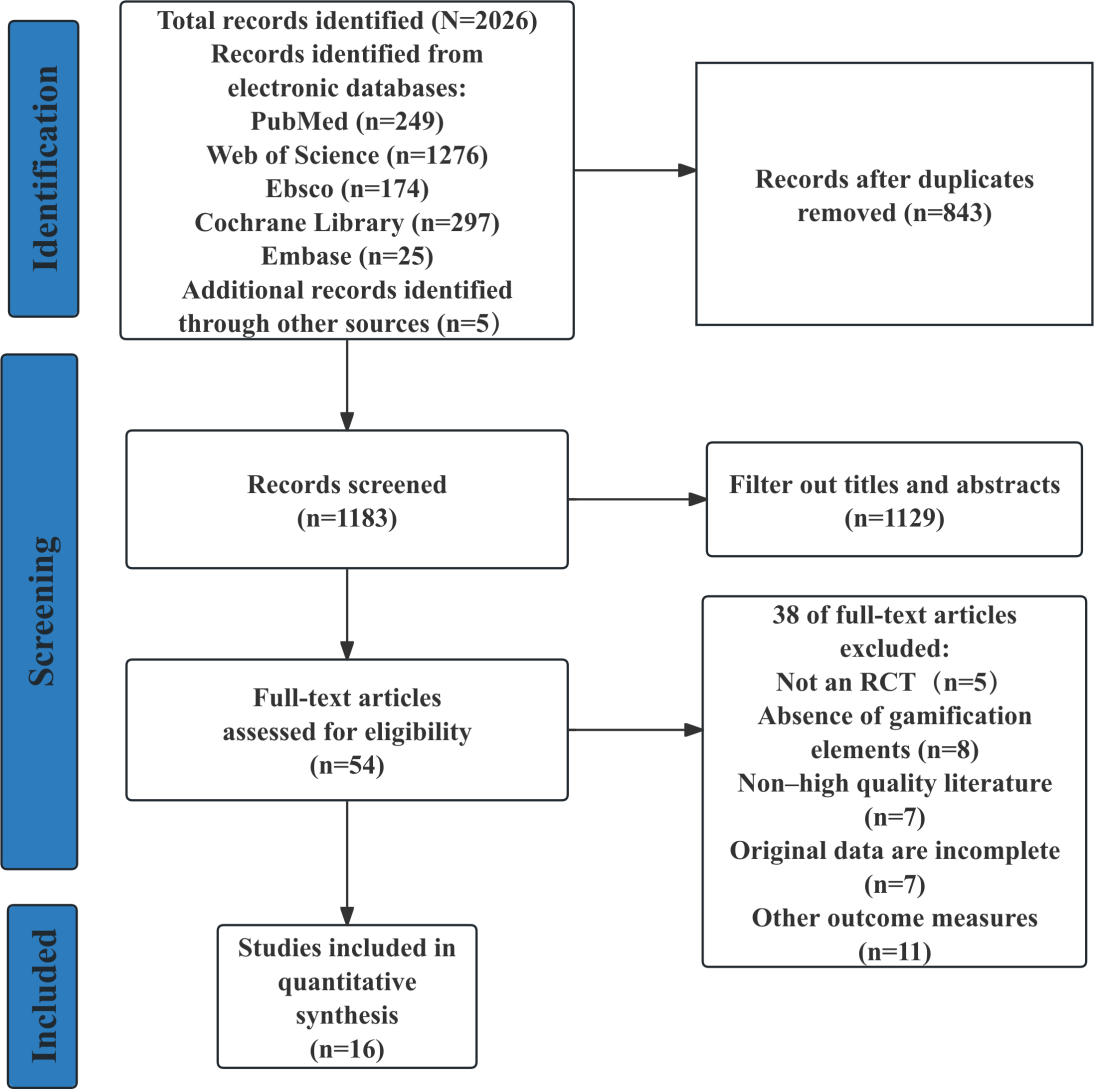
Flowchart of the study selection. RCT: randomized controlled trial.

A total of 2021 relevant articles were retrieved from PubMed (n=249), Web of Science (n=1276), EBSCO (n=174), Cochrane Library (n=297), and Embase (n=25), and 5 records were retrieved from the supplementary register. Figure 1 illustrates the study selection workflow. After removing 843 duplicate records using EndNote 21, 1183 unique records underwent title and abstract screening. This process excluded 1129 irrelevant publications, yielding 54 articles for full-text assessment. Following full-text review, 38 articles were excluded due to predefined criteria (eg, non-RCT design and irrelevant outcomes), resulting in 16 eligible studies included for final analysis [41-56].

### Characteristics of the Included Studies

The 16 English-language papers included in this study were published from 2012 to 2024 and contained 7472 subjects, ranging from 13 to 1544 subjects per study [41-56]. The included studies had the following essential characteristics. Participants ranged in age from 1 to 18 years. Study countries included the United States (5/16) [45,50,53-55], Canada (4/16) [46-48,56], New Zealand (2/16) [43,44], Finland (1/16) [49], Chile (1/16) [51], Northern Ireland (1/16) [42], Singapore (1/16) [52], and the United Kingdom (1/16) [41]. A total of 9 studies were tested in school settings [41,42,44-47,51,53,54], 5 in home settings [50,52,53,55,56], and 3 in settings where families and communities worked together [43,48,49]. Furthermore, 12 studies were RCTs [43,45-50,52-56] and 4 studies were cluster RCTs [41,42,44,51]. In addition, 7 studies used belt-worn accelerometers [43-45,50,53-55], 5 studies used wristband activity trackers [41,42,49,51,56], 3 studies used foot sensors as activity trackers [46-48], and 1 study used the MapMyFitness app (Under Armour) on a smartphone, eliminating the need for an additional wearable activity tracker [52]. The intervention period was 1‐48 weeks, and the follow-up was 24‐96 weeks. Outcome measures included MVPA, VPA, MPA, LPA, SB, and daily steps. A total of 14 studies used behavior change research theories, including SCT [53,55], SDT [41,42,45-48,52,54], behavior change wheel (BCW) [51], BCT [43], and TTM [49,56]. The trials have been registered in the Clinical Trials Registry [41-51,53-56], and 1 trial is not registered [52]. Furthermore, 14 studies have been funded [41-44,46-51,53-56] and 2 studies did not receive any funding [45,52]. In addition, 1 completed follow-up 48 to 96 weeks (mean 12 weeks) after completing the intervention [44], and 3 studies completed follow-up assessments 12‐28 weeks after the intervention ended [42,51,55]. In terms of how gamification interventions were implemented, 14 studies used mobile apps (eg, smartphone apps, iPod touch, Facebook groups, and Fitbit mobile apps) in addition to wearable PA trackers [41-43,45-50,52-56], and 2 studies focused on building school gamification environments [44,51]. The number of game elements used in gamification interventions ranged from 1 to 8, with most including 4 or more game elements. The most used game elements were socialization, interaction, rewards, points, feedback, challenges, and achievements. In addition, intervention durations ranged from 1 week to 96 weeks, with 5 (31.3%) studies having a follow-up period. This suggests that further evaluation of PA gamification is needed to determine the effectiveness and sustainability of game element interventions (Table 1).

**Table 1. T1:** Summary of the intervention characteristics of the included studies.

Study	Sample size		Participants (age range in years)	Interventions	Comparator	Theoretical paradigm	Duration (weeks)	Game elements	Main outcomes	Country
	N	Male, n (%)								
Maloney et al [50], 2012	64	53 (82.8)	Adolescents with obesity or overweight (9-17)	Pedometer plus DDR (‘Dance Revolution’)	Pedometer only	No theory mentioned	12	Interaction	VPA^a^, MPA^b^, LPA^c^, and daily steps	The United States
Garde et al [48], 2015	47	16 (34)	Adolescents (8-13)	“Mobile Kids Monster Manor “was a mobile exergame synchronized with an external activity monitor	Feedback through tractility	SDT^d^	2	Points, rewards, feedback, social interaction, challenges, and achievements	Daily steps	Canada
Garde et al [47], 2016	56	35 (62)	Adolescents (10-13)	“Mobile Kids Monster Manor (MKMM) combines a game with activity monitors.”	No intervention	SDT	1	Points, rewards, feedback, social interaction, challenges, and achievements	Daily steps	Canada
Garde et al [46], 2018	37	16 (43)	Adolescents (9-11)	“MobileKids Monster Manor (MKMM) combines a game with activity monitors.”	No intervention	SDT	1	Points, rewards, feedback, social interaction, challenges, and achievements	Daily steps	Canada
Fu et al [45], 2018	65	34 (52)	Preschool children (4-6)	“Go Noodle” is an online active video game program that provides children with a variety of PA and exercise choices	30 min active free-play sessions	SDT	12	Social interaction	Daily steps	The United States
Staiano [53], 2018	46	23 (50)	Children with obesity or overweight (10-12)	Exergaming group (Kinect and Xbox 360 gaming console, exergames, telehealth coaching)	No intervention	SCT^e^	24	Social interaction	zBMI^f^	The United States
Staiano et al [54], 2016	42	0 (0)	Adolescent girls (14-18)	Dance-based motion game intervention, featuring a structured gaming environment	No intervention	SDT	12	Social interaction	SB^g^, LPA, MPA, and VPA	The United States
Pena et al [51], 2021	2320	1398 (60.2)	Students (10-12)	Nutrition and physical activity interventions using gamification strategies	Standard education curriculum	BCW^h^	16‐28	Points, leaderboards, badges, and rewards	zBMI	Chile
Corepal et al [42], 2019	224	105 (46)	Adolescents (12-14)	Step Smart Challenge (Basic pedometer competition for schools, including gamified strategies)	No intervention	SDT	22‐52	Feedback and rewards	MVPA^i^, LPA, VPA, MPA, SB, and daily steps	Northern Ireland
Leinonen et al [49], 2017	496	496 (100)	Adolescents (17-18)	The intervention group used a gamified application, web-based mobile body and social activation service	No intervention	TTM^j^	24	Challenges, rewards, and competition	MVPA	Finland
Corder et al [41], 2020	2862	1491 (52)	Adolescents (13-14)	Go Active intervention and gained points and rewards	Regular school activities	SDT	40	Point	MVPA	The United Kingdom
Direito et al [43], 2015	51	22 (43)	Adolescents (14-17)	Use an immersive app (Zombies, Run) and a nonimmersive app (Get Running).	Regular behavior group	BCTs	8	Points and challenges	MVPA, VPA, MPA, LPA, and SB	New Zealand
Tugault-Lafleure et al [56], 2023	214	104 (48.5)	Adolescents (10-17)	Use the Aim2Be app and get guidance from in-person coaches.	Only accept guidance from regular monitoring	TTM	12	Storytelling and narrative	TPA, zBMI, SB, and daily steps	Canada
Seah et al [52], 2020	36	0 (0)	High school girls (14-16)	Use the MapMyFitness (MMF) mobile app to track your workouts and get feedback.	Regular weekend activities	SDT	4	Tasks and challenges, rewards (currency earning and unlockable rewards), and social features	Daily steps	Singapore
Farmer et al [44], 2017	840	422 (50.2)	Children (1-8)	Redesigned play environments to encourage imaginative and independent free play	No intervention	1	48‐96	Feedback, incentives, and social features	MVPA and zBMI	New Zealand
Staiano et al [55], 2022	72	31 (43)	Children (3-5)	Motor Skills app: instructional lessons, peer modeling videos, and behavioral scaffolding	Free Play app: lessons and videos promoting	SCT	12‐24	Social interaction	MVPA, LPA, and SB	The United States

a VPA: vigorous physical activity.

b MPA: moderate physical activity.

c LPA: light physical activity.

d SDT: self-determination theory.

e SCT: social cognitive theory.

f zBMI: BMI *z*-score.

g SB: sedentary time or behavior.

h BCW: behavioral change wheel.

i MVPA: moderate-to-vigorous physical activity.

j TTM: the transtheoretical model.

### Risk of Bias Assessment

**Figure 2.**
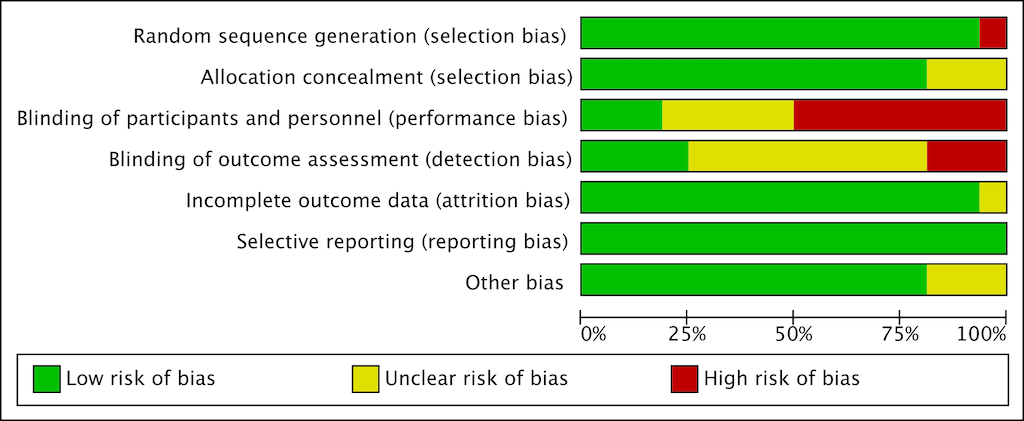
Risk of bias graph.

The *κ* coefficient of the risk of bias data extracted by both authors was 0.73, which is in excellent agreement. The authors’ ratings for each risk of bias item are presented as percentages in the meta-analysis, and the different biases for each study are summarized. Overall, 15 trials adequately reported randomized sequence generation, and 1 trial with randomized sequence generation was considered high risk because the trials were assigned sequentially in the order of recruitment [48], 13 trials (81%) described programs designed to conceal [41,43,45-53,55,56]. The blinding of the subjects and the personnel in a total of 5 trials is not known [41,46,49,52,53], while 8 trials had unclear blinding of the outcome assessments [43,44,46,47,49,50,52,54]. A total of 15trials provided data on subjects lost to follow-up. In contrast, 1 trial had unclear information [54], and 2 trials were at high risk of other bias [42,52]. In addition, 1 crossover trial was conducted without a blinding condition [47], and 1 trial has not yet been registered for publication, so there is uncertainty [56] (Figures2  3).

**Figure 3.**
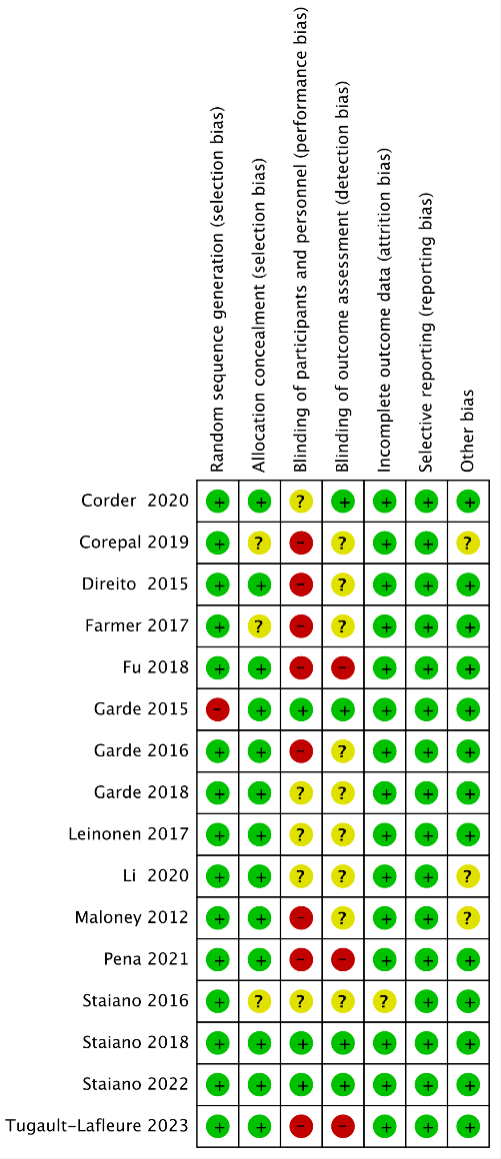
Risk of bias summary [41-56].

### Results of the Meta-Analysis

**Figure 4.**
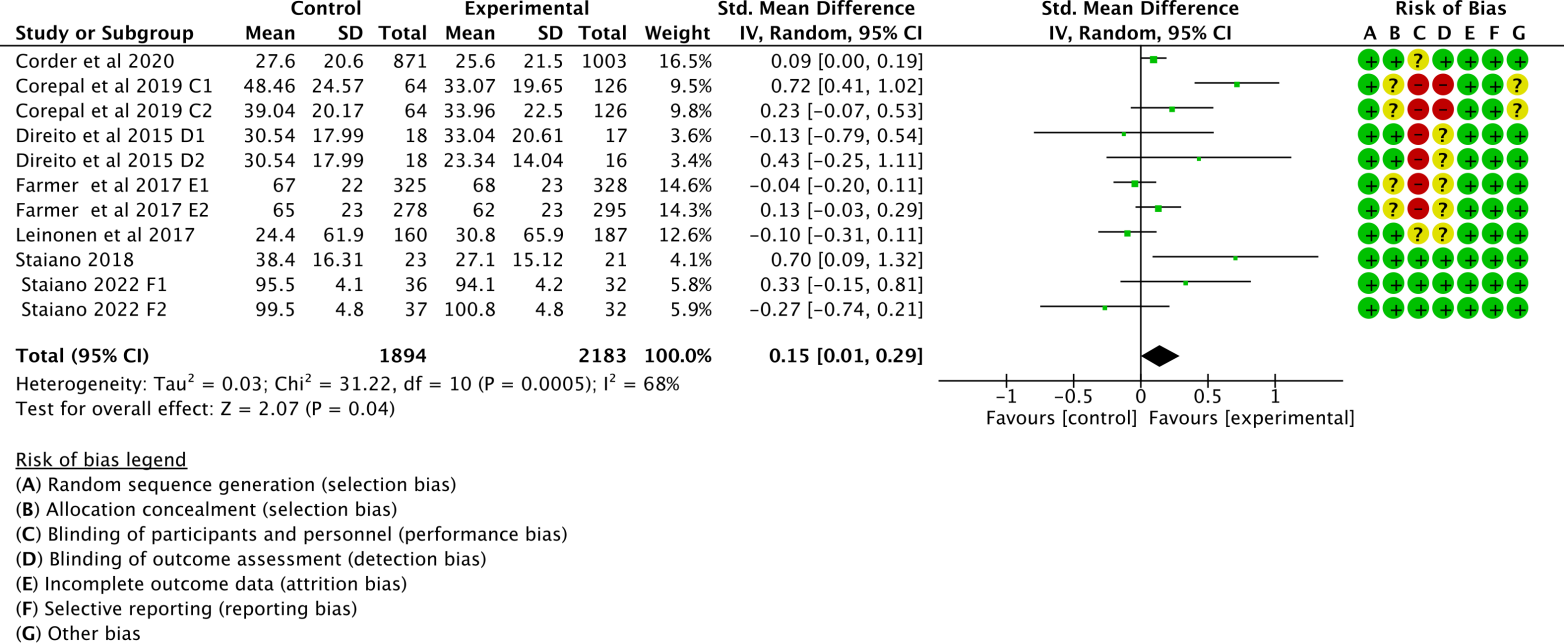
Forest plot of the effect of gamification interventions on increasing moderate to vigorous physical activity [41-44,49,53,55].

Of the 16 studies, 11 (69%) were examined to assess the impact of gamification interventions on MVPA. The heterogeneity test showed a significant heterogeneity among the studies (*I*^2^=68%; *P*<.1), so a random effects model was used for the analysis. The combined effect sizes showed that the gamification intervention significantly improved MVPA in children and adolescents compared to the control group (SMD 0.15, 95% CI 0.01 to 0.29; *P*=.04); see Figure 4 [41-44,49,53,55].

**Figure 5.**
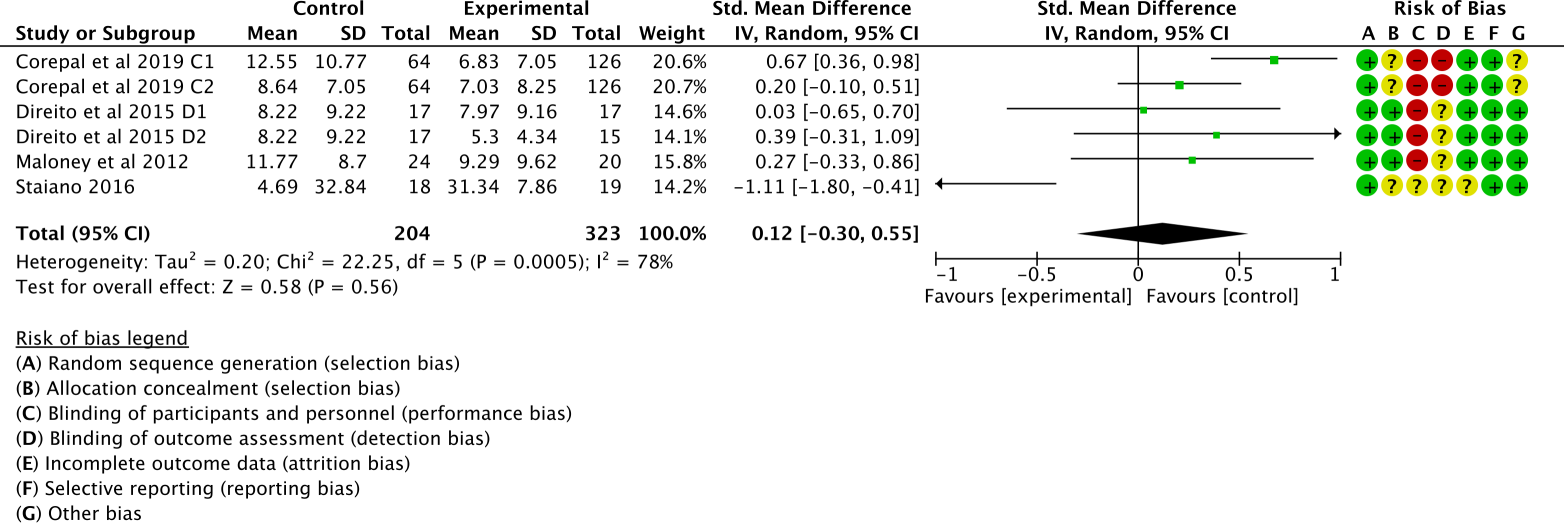
Forest plot of the effect of gamification interventions on increasing vigorous physical activity [42,43,50,54].

Of the 16 studies, 6 (38%) were examined to assess the impact of gamification interventions on VPA. The heterogeneity test revealed heterogeneity between studies (*I*^2^=78%; *P*<.001), which required analysis using a random effects model. The combined effect size indicated that gamification interventions were ineffective in improving VPA in children and adolescents compared to controls (SMD 0.12, 95% CI −0.3 to 0.55; *P*=.56); see Figure 5 [42,43,50,54].

**Figure 6.**
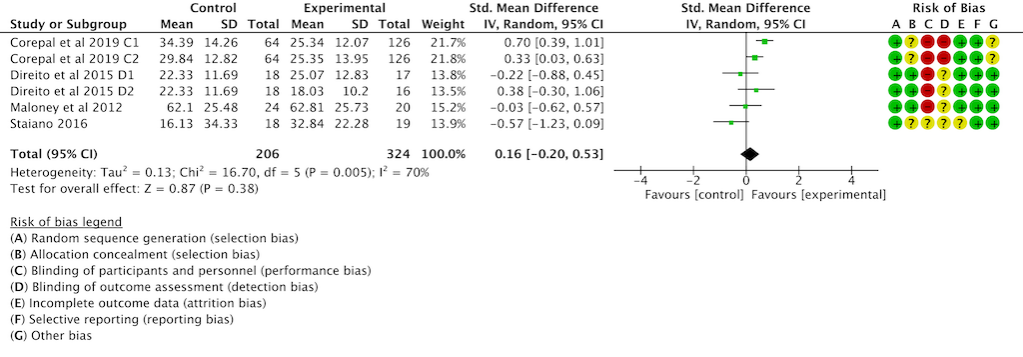
Forest plot of the effect of gamification interventions on increasing moderate physical activity [42,43,50,53].

Of the 16 studies, 6 (38%) were examined to assess the impact of gamification interventions on MPA. Tests for heterogeneity showed more significant heterogeneity between studies (*I*^2^=70%; *P*<.001), requiring analysis using random effects models. The meta-analysis found no significant differences in the effects of gamification interventions on MPA in children and adolescents compared with controls (SMD 0.16, 95% CI −0.2 to 0.53; *P*=.38); see Figure 6 [42,43,50,53].

**Figure 7.**
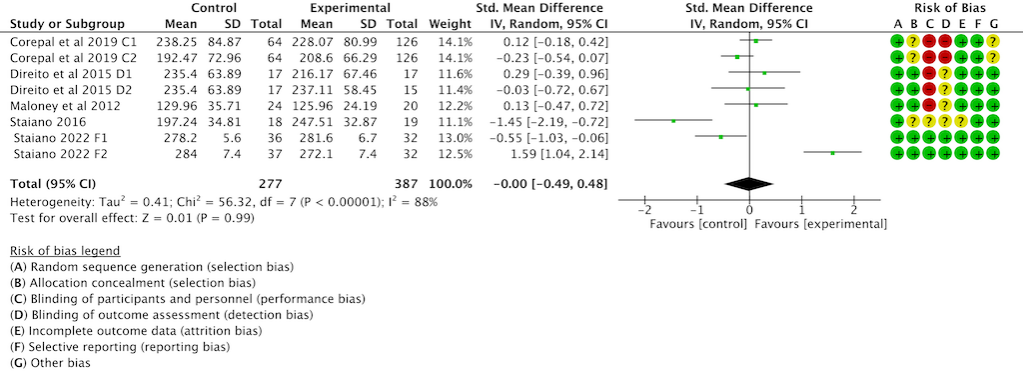
Forest plot of the effect of gamification interventions on increasing light physical activity [42,43,50,54,55].

Of the 16 studies, 8 (50%) were examined to assess the impact of gamification interventions on LPA. The heterogeneity test revealed heterogeneity between studies (*I*^2^=88%; *P*<.001), which required analysis using a random effects model. The combined effect sizes showed that the difference in LPA between the gamification intervention and the control group was insignificant (SMD −0.00, 95% CI −0.49 to 0.48; *P*=.99); see Figure 7 [42,43,50,54,55].

**Figure 8.**
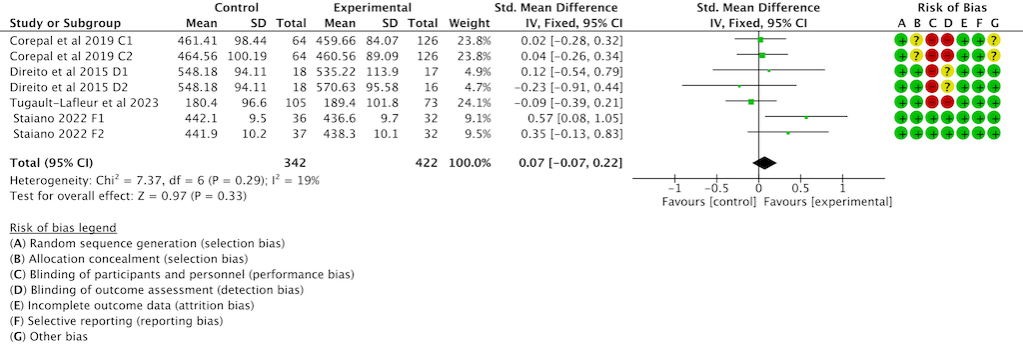
Forest plot of the effect of gamification interventions on decreasing sedentary behavior [42,43,55,56].

Of the 16 studies, 7 (44%) were examined to assess the impact of gamification interventions on SB. Heterogeneity tests showed heterogeneity between studies (*I*^2^=19%; *P*>0.01), so fixed effects models were used for analyses. A meta-analysis found that gamification interventions did not significantly reduce SB in children and adolescents (SMD 0.07, 95% CI −0.07 to 0.22; *P*=.33); see Figure 8 [42,43,55,56].

**Figure 9.**
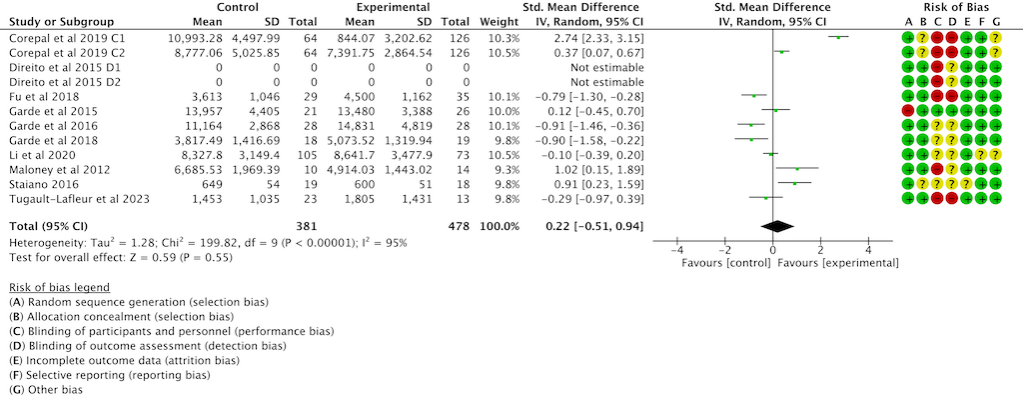
Forest plot of the effect of gamification interventions on daily steps [42,45-48,50,52,54,56].

Of the 16 studies, 12 (75%) were examined to assess the impact of gamification interventions on daily step counts. Tests for heterogeneity showed more significant heterogeneity between studies (*I*^2^=95%; *P*<.001), requiring analysis using random effects models. A meta-analysis found that gamification interventions did not increase daily steps among children and adolescents (SMD 0.22,95% CI −0.51 to 0.94; *P*=.55); see Figure 9 [42,45-48,50,52,54,56].

**Figure 10.**
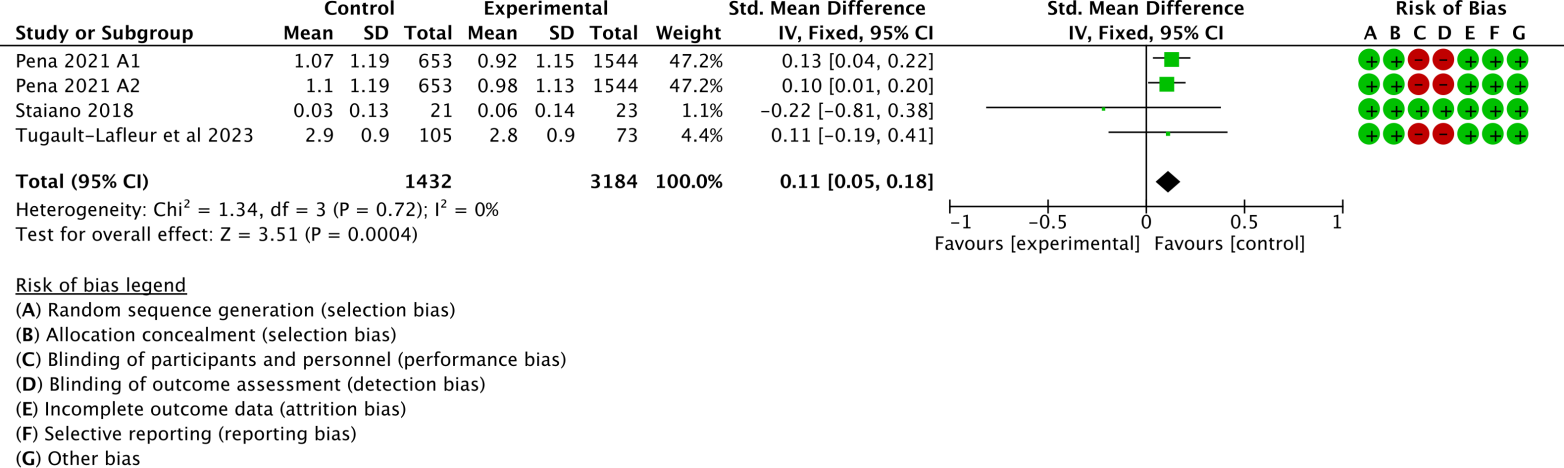
Forest plot of the effect of gamification interventions on BMI [51,53,56].

Of the 16 studies, 4 (25%) were examined to assess the impact of gamification interventions on BMI. Heterogeneity tests showed little heterogeneity between studies (*I*^2^=0%; *P*=.72), which required analysis using a fixed-effects model. A meta-analysis found that gamification interventions improved BMI in children and adolescents (SMD 0.11, 95% CI 0.05 to 0.18; *P*<.001); see Figure 10 [51,53,56].

### Sensitivity Analysis

In this study, sensitivity analyses of MVPA, LPA, and SB were conducted using Stata 18.0 to assess the robustness and reliability of the results. The sensitivity analyses showed that excluding any of the studies did not affect the magnitude of the effect of the gamification intervention on the outcomes of LPA (Multimedia Appendix 2) and SB (Multimedia Appendix 3), suggesting the robustness and reliability of the findings [12]. However, for MVPA (Multimedia Appendix 4), the sensitivity analysis identified 4 studies as outliers [41,42,53,55]. Excluding one of these studies changed the overall effect size, suggesting that the results were not robust enough and should be interpreted cautiously.

### Subgroup Analyses

To further explore the effects of other components on outcome indicators, this study focused on subgroup analyses of MVPA, including age, theoretical paradigm, game elements, intervention duration, study setting, position of the PA monitor, and sample size. Only 4 articles assessed BMI as an outcome. Subgroup analyses were not performed for this outcome due to the small sample size. In addition, the results of all subgroup analyses for SB and other PA indicators were not significant and were, therefore, not analyzed in detail.

### A Subgroup Analysis of the Effects of a Gamification Intervention on MVPA

The results of the subgroup analyses of the gamification intervention on MVPA are presented in Multimedia Appendix 5, and there were no significant differences in age, wearer position, or sample size regarding improvements in MVPA in children and adolescents. The theoretical paradigm, intervention during, and game elements significantly moderate the effectiveness of gamification interventions in enhancing MVPA. Subgroup analyses based on theoretical paradigms showed a more pronounced effect of SDT-based interventions on MVPA promotion (SMD 0.39, 95% CI 0.01 to 0.77; *I*^2^=91%). However, the effect of other theoretical paradigms based on SCT (SMD −0.19, 95% CI −0.76 to 0.39; *I*^2^=72.6%) and BCTs (SMD 0.01, 95% CI −0.11 to 0.13; *I*^2^=25%) was negligible. Subgroup analyses based on game elements showed significantly larger effect sizes for interventions based on elements such as rewards and feedback (SMD 0.19, 95% CI 0.01 to 0.37; *I*^2^=86%) compared to interventions based primarily on social interaction (SMD −0.07, 95% CI −0.46 to 0.33; *I*^2^=58%), with more significant heterogeneity between the two groups, but effect sizes 95% CI overlapped. Subgroup analyses based on intervention duration showed a more significant MVPA promotion effect for interventions >12 weeks compared to interventions ≤12 weeks (SMD 0.02, 95% CI −0.15 to 0.19; *I*^2^=76.9% and SMD 0.14, 95% CI 0.02 to 0.26; *I*^2^=1.1%), less heterogeneity. There was no overlap in the 95% CIs of effect sizes. Subgroup analyses based on trial setting showed that school-based interventions (SMD 0.18, 95% CI 0.01 to 0.36; *I*^2^=79%) had larger effect sizes than home-based or combined home- and community-based interventions (SMD −0.08, 95% CI −0.35 to 0.20; *I*^2^=48%). Heterogeneity was also more significant, and there was no overlap in the 95% CI for effect sizes.

### Reporting Biases

Qualitative and quantitative methods were used to assess publication bias. Funnel plots were used to assess publication bias for the effects of gamification interventions on MVPA, LPA, and SB in children and adolescents. Funnel plots showed a mostly symmetrical pattern across the 3 studies (Multimedia Appendices 6-8). The Egger test was used to assess the size of publication bias of the included studies for all 3 studies (Multimedia Appendices 9-11). The Egger test was performed for MVPA (*t*_11_=0.05; *P*=.97), LPA (*t*_8_=0.01; *P*=.99), and SB (*t*_7_=–0.83; *P*=.44). Therefore, all the literature included was judged to be free of publication bias in a comprehensive manner.

## Discussion

### Principal Findings

This paper systematically reviews the effects of gamification interventions on MVPA, VPA, MPA, LPA, SB, daily steps, and BMI in children and adolescents through a meta-analysis of 16 RCTs. It also responds to the call for future research needs by Mazeas et al [15]. The absence of significant publication bias for all outcomes in the included articles enhances the reliability of the evidence. The results showed that the gamification intervention group had a positive effect on improving MVPA but a more minor effect on reducing SB than the control group. Age, theoretical paradigm, game elements, and intervention duration were identified as essential moderators associated with the increased effectiveness of gamification interventions on MVPA. However, the effects on effect sizes were not entirely consistent.

### Impact of Gamification Interventions on PA

The study found that the gamification intervention significantly increased MVPA in children and adolescents but did not show positive effects on VPA, MPA, LPA, daily steps, and reduction in SB, with an overall small, combined effect size. Although our findings are an extension of the most recently published systematic review, they are inconsistent with previous findings [15]. Previous research has demonstrated that gamification interventions can increase levels of PA and have shown efficacy in promoting PA in nonhealthy individuals as well as those with chronic disease, particularly in adults and older adults, as evidenced by significant increases in steps per day and low-intensity PA [57,58]. This increase was particularly evident in studies of adults with overweight or obesity, cancer survivors, and patients with chronic cardiometabolic disease [58-60]. In addition, people who are overweight or obese may have increased their PA levels because of weight loss goals or specific weight loss programs. However, compared with these studies, the results showed more minor and nonsignificant effects with lower quality of evidence. This may be because increasing PA in children and adolescents has proven challenging, whether through home-based gamification interventions or interventions targeting the school environment or curriculum [27,61,62]. This reduces individual adherence to some extent [60]. A total of 6 studies in the included literature showed poor adolescent adherence during the intervention, which affected the final study results [42,46-48,55,56]. Alternatively, the lack of benefit we observed for the intervention may be because part of the target population was already sufficiently active, which leaves little room for an increase in MVPA [44]. In addition, 5 of these studies had baseline activity levels greater than 150 min/d [42,43,45,48,55]. The paper also found daily step goal orientation essential for promoting increased daily step counts [63]. Research has shown that setting higher PA goals typically motivates individuals to achieve higher PA [64,65]. This effect is powerful when combined with goal setting, self-monitoring, and rewarding feedback [66]. Interventions targeting step goal setting were more limited in the included literature. This further explains the limited role of gamification interventions in promoting daily steps and low-intensity PA in healthy adolescents. At the same time, given the limited amount of relevant literature on both, the validity of this finding requires further validation.

MVPA subgroup analyses indicated that interventions using reward-feedback game mechanics (eg, points systems and performance badges) demonstrated superior efficacy. Compared to interventions emphasizing social interaction elements, reward-based approaches significantly enhanced PA engagement (SMD 0.19, 95% CI 0.01 to 0.37; *P*=.04). Intervention duration and theoretical frameworks emerged as critical moderators: Long-term interventions (>12 weeks) yielded substantially greater MVPA (SMD 0.14, 95% CI 0.02 to 0.26; *P*=.02) improvement than short-term programs (≤12 weeks), confirming the sustainability of gamification effects and aligning with existing evidence on behavioral maintenance [15]. Theoretical paradigms guided 88% (14/16) of the included studies. SDT and SCT were the predominant frameworks, consistent with previous systematic reviews highlighting their use in PA interventions [67]. SDT is a well-established theory of motivation that has become a critical framework for health behavior interventions. An individual’s motivation, considered the primary driver of behavior change, is often viewed as an extrinsic driver of PA activity. While elements such as rewards and feedback, primarily goal-directed, are considered intrinsic drivers, it is difficult to distinguish whether the effect is due to the sum of the internal and external drivers or an independent driver intervention. Subgroup analyses in this study indicated that game elements such as rewards and feedback can promote MVPA and that interventions from the SDT theoretical paradigm are effective in promoting MVPA. However, due to the limited amount of literature included, it was not possible to explore the importance of combining both on PA further. Therefore, further coordinated interventions are recommended for the future, such as increasing the weight of elements such as rewards and feedback in gamification while synergizing the SDT theoretical paradigm.

### Impact of Gamification Interventions on SB

Meta-analysis revealed that gamification interventions failed to significantly reduce SB (SMD 0.07,95% CI −0.07 to 0.22; *P*=.33) in children and adolescents, with low-quality evidence aligning with previous studies [68,69]. Critically, this nonsignificant outcome more likely reflects methodological constraints rather than intervention inefficacy. Key evidence includes: (1) Pivotal moderating role of family environment: Family settings served as the primary context for gamification interventions [70,71], with 5 out of 7 included studies (71%) implemented in home environments. For instance, a Canadian eHealth intervention study demonstrated that family environment—particularly parenting styles, caregiving approaches, and household income—predicted a substantial proportion of variance in adolescents’ intervention adherence [72]. Tugault-Lafleur and colleagues’ [56] data also confirmed a strong relationship between parental adherence and youth participation. Only 1 trial [42] in the included literature assessed sedentary behavior in a school setting, and due to the small sample size, subgroup analyses of this outcome were not conducted. (2) Limitations in measurement tool adherence: Accelerometer-based PA monitoring demonstrated suboptimal participant adherence [73], while variations in wear time significantly biased SB estimates [74]. Notably, 2 included studies reported a decline in accelerometer adherence during interventions [43,44], with no intergroup differences (*P*>.05), potentially compromising outcome validity. (3) Efficacy gap in intervention design; Interventions targeting SB are more effective than interventions promoting PA [75]. Paradoxically, most included studies (4/7) lacked explicit SB-reduction objectives or personalized strategies [43,49,51,53]. Although 2 studies incorporated SB-focused components [42,52], these relied on passive advisory methods (eg, Facebook groups or VFB ecosystems). Such approaches likely failed to engage adolescents’ active attention to device-generated feedback and elicit timely behavioral responses.

### Moderating Variables on the Effects of Gamification Interventions for PA

#### Theoretical Paradigm

Several types of BCTs are currently commonly used in gamification interventions, and they have also been shown to be effective in influencing intervention outcomes [42]. Only 2 of 16 studies (12.5%) did not use a theoretical paradigm in their gamification interventions [44,50]. The majority was SDT (8/16). SDT is the most used theoretical paradigm in gamification intervention trials. SDT-based interventions are effective in improving MVPA (SMD 0.39, 95% CI 0.01 to 0.77; *P*=.04), in children and adolescents [23]. SDT is a well-established theory of motivation that has become a critical framework for health behavior interventions because of the belief that an individual’s motivation is the primary driver of behavior change [76]. However, intrinsic or extrinsic motivation has different effects on behavior change. Existing research suggests that intrinsic motivation promotes more stable behavior change and psychological and social well-being [23]. Therefore, future research could use gamification to promote intrinsic motivation and increase PA engagement. Second, SCT is another theoretical framework widely adopted in PA promotion interventions. SCT proposes that a combination of personal factors, the social environment, and the physical environment influences an individual’s level of PA. Among these factors, self-efficacy, self-regulation, and efficacy expectations are vital personal factors that promote PA and reduce SB [77]. In conclusion, SCTor SDT-based interventions may produce more effective outcomes than gamification interventions. However, due to the limited number of included studies, further validation through many high-quality studies is needed to support these conclusions.

#### Intervention Duration

Subgroup analysis of intervention duration showed an association between the effect of gamification on MVPA and intervention duration. Compared to intervention ≤12 weeks, intervention cycles of more than 12 weeks (including the follow-up period) were more likely to promote MVPA (SMD 0.14, 95% CI 0.02 to 0.26; *P*=.02), with a mean duration of 32.67 weeks (SD 25.8 weeks), suggesting that the effects of gamification persisted beyond the end of the intervention. Smartphone apps have been claimed to have a significant positive effect on PA only when used for a short period of <3 months [46], which is inconsistent with the current meta-analysis results. This may be due to the playful nature of gamification, where the effectiveness of the intervention is not only influenced by the intervention period but also by age, theoretical paradigm, game elements, and the type of measurement tool. It is worth noting that gamification interventions are not just a novelty effect and should have some long-term effects. However, given the limited amount of literature included, it is still worth exploring whether this long-term effect diminishes after the intervention has ended.

#### Game Elements

Achievement-based and goal-oriented game elements, such as rewards and feedback, were the most frequently used game elements in the systematic evaluation, consistent with the results of the previous evaluation [36,78]. Analyses of the PA subgroup showed that a gamification intervention with rewards and feedback at its core significantly increased MVPA, effectively combining techniques to promote behavior change and motivate participants to be active. However, it has also been argued that extrinsic motivation (rewards, etc) may be detrimental to the long-term maintenance of intervention effects compared to intrinsic motivation (SDT or SCT) [79]. The effectiveness of socialization and interactivity, the second most used game element, is significantly influenced by the type of social incentive and the application method [80]. Gamification strategies significantly increased PA levels when used collaboratively between families. In contrast, they were less effective when used with individuals who did not know each other as intervention targets [80]. Therefore, future studies must explore the effectiveness of gamification interventions that integrate different social incentives to increase PA engagement and assess the applicability and impact of these interventions in different social relationship contexts. This will help to develop more precise and personalized gamified health promotion programs.

### Limitations

This study has several limitations. First, the included literature was in English, which may have led to selection bias, and some studies had small sample sizes and poor compliance, which increased the risk of publication bias. Second, due to the nature of the intervention, the included literature did not implement blinding in data processing and analysis, as well as differences in recruitment settings, which may affect the results of the meta-analysis. Third, the outcome indicators used in the included literature studies had inconsistent units, and using SMD as an effect indicator requires caution in interpreting the results. Results suggest heterogeneity in some studies, and the source of this heterogeneity is unclear. The effectiveness of gamification interventions may vary among children and adolescents depending on demographics (eg, ethnic background, region, gender, and BMI), economic level, type of intervention (parent-centered vs child-centered), and degree of application personalization. These factors may have contributed to the observed heterogeneity. However, these factors were not analyzed in this study due to limitations imposed by the number and characteristics of the included studies. In conclusion, the results of this systematic review and meta-analysis should be interpreted with caution.

### Conclusions

Gamification interventions effectively increase MVPA in children and adolescents, and this effect persists beyond the follow-up period. This suggests that the effects of gamification interventions are not solely due to their novelty but have long-term effects. However, the effects of gamification interventions on improving VPA, MPA, LPA, daily step count, and SB are not yet apparent. The number and quality of studies limit them and should be interpreted cautiously. In addition, theoretical paradigms, game elements, and intervention duration may be associated with the effectiveness of gamification interventions, and further research is needed on the optimal implementation of game elements and theoretical features to maximize PA engagement. In conclusion, due to the limited number and quality of included studies, the above findings need to be validated by additional high-quality studies.

## Supplementary material

10.2196/68151 Multimedia Appendix 1 Literature search strategy.

10.2196/68151 Multimedia Appendix 2 Sensitivity analyses results on light physical activity.

10.2196/68151 Multimedia Appendix 3 Sensitivity analyses result on sedentary behavior.

10.2196/68151 Multimedia Appendix 4 Sensitivity analyses result on moderate-to-vigorous physical activity.

10.2196/68151 Multimedia Appendix 5 Summary of subgroup analysis results of gamification interventions for moderate-to-vigorous physical activity.

10.2196/68151 Multimedia Appendix 6 Funnel plot of moderate-to-vigorous physical activity.

10.2196/68151 Multimedia Appendix 7 Funnel plot of sedentary behavior.

10.2196/68151 Multimedia Appendix 8 Funnel plot of light physical activity.

10.2196/68151 Multimedia Appendix 9 Egger test results for light physical activity.

10.2196/68151 Multimedia Appendix 10 Egger test results for sedentary behavior.

10.2196/68151 Multimedia Appendix 11 Egger test results for moderate-to-vigorous physical activity.

10.2196/68151 Checklist 1 PRISMA 2020 checklist.

## Abbreviations

BCT: behavior change technique
BCW: behavior change wheel
LPA: light physical activity
MPA: moderate physical activity
MVPA: moderate-to-vigorous physical activity
PA: physical activity
PRISMA: Preferred Reporting Items for Systematic Reviews and Meta-Analyses
PROSPERO: International Prospective Register of Systematic Reviews
SB: sedentary behavior
SCT: social cognitive theory
SDT: self-determination theory
SMD: standardized mean difference
TTM: transtheoretical model
VPA: vigorous physical activity
zBMI: BMI z-score
