# Supplementary material for: Effectiveness of Gamification Interventions to Improve Physical Activity and Sedentary Behavior in Children and Adolescents: Systematic Review and Meta-Analysis
Source: JMIR Serious Games. 2025 Sep 18;13:e68151. doi: 10.2196/68151 (PMC12445784; doi:10.2196/68151)
Supplement: Multimedia Appendix 1 [file games-v13-e68151-s001.doc]

**Multimedia Appendix 1: Literature search strategy**

**Database searched: PubMed (249)**

#1. ((((((((((((("Adolescent"[Mesh]) OR (Adolescents[Title/Abstract])) OR (Adolescence[Title/Abstract])) OR (Female Adolescent[Title/Abstract])) OR (Female Adolescents[Title/Abstract])) OR (Male Adolescent[Title/Abstract])) OR (Male Adolescents[Title/Abstract])) OR (Youth[Title/Abstract])) OR (Youths[Title/Abstract])) OR (Teens[Title/Abstract])) OR (Teen[Title/Abstract])) OR (Teenagers[Title/Abstract])) OR (Teenager[Title/Abstract])) OR ((((((("Child"[Mesh]) OR (Children[Title/Abstract])) OR (Child[Title/Abstract])) OR (Preschool[Title/Abstract])) ) OR (Preschool Child[Title/Abstract])) OR (Preschool Children[Title/Abstract]))

#2. ((((((((((((((((((("Exercise"[Mesh]) OR (Physical activity[Title/Abstract])) OR (Exercise[Title/Abstract])) OR (Physical Exercise[Title/Abstract])) OR (Physical Activity[Title/Abstract])) OR (Physical Activities[Title/Abstract])) OR (Aerobic Exercise[Title/Abstract])) OR (Isometric Exercise[Title/Abstract])) OR (Acute Exercise[Title/Abstract])) OR (Exercise Training[Title/Abstract])) OR (Exercise Trainings[Title/Abstract])) OR (PA[Title/Abstract])) OR (moderate to vigorous physical activity[Title/Abstract])) OR (moderate-to-vigorous physical activity[Title/Abstract])) OR (leisure time activity[Title/Abstract])) OR (leisure-time activity[Title/Abstract])) OR (walking[Title/Abstract])) OR (steps[Title/Abstract])) OR (running[Title/Abstract])) OR ((((((((("Sedentary Behavior"[Mesh]) OR (Sedentary behavior[Title/Abstract])) OR (Sedentary Behaviors[Title/Abstract])) OR (Sedentary Lifestyle[Title/Abstract])) OR (Physical Inactivity[Title/Abstract])) OR (Lack of Physical Activity[Title/Abstract])) OR (Sedentary Time[Title/Abstract])) OR (inactive[Title/Abstract])) OR (inactivity[Title/Abstract]))

#3. ((("Gamification"[Mesh]) OR (gamification [Title/Abstract])) OR (exergaming [Title/Abstract])) OR (exer-gaming[Title/Abstract])

#4. (((((("Randomized Controlled Trial" [Publication Type]) OR (Randomized Controlled Trial [Title/Abstract])) OR (Random control test [Title/Abstract])) OR (controlled clinical trial[Title/Abstract])) OR (Randomized[Title/Abstract])) OR (Randomly[Title/Abstract])) OR (Trial[Title/Abstract])

#5. #1 AND #2 AND #3 AND #4

#6. (((( (((((((game[Title/Abstract]) OR (games[Title/Abstract])) OR (gamified[Title/Abstract])) OR (gaming[Title/Abstract])) OR (gameful*[Title/Abstract])) OR (multiplayer[Title/Abstract])) OR (player[Title/Abstract])) OR (playing[Title/Abstract])) OR (Challenges[Title/Abstract])) OR (checkpoint[Title/Abstract])) OR (Rewards[Title/Abstract])) OR (Combine game elements[Title/Abstract])

#7. #1 AND #2 AND #4 AND #6

#8. #5 OR #7

Filters: English, from 2010-2024

**Database searched: Web of Science Core Collection (1276)**

#1. ((TS=(Child) OR AB=(Children OR Child OR Preschool OR Preschool Child OR Preschool Children))OR (TS=(Adolescent) OR AB=(Adolescents OR Adolescence OR Female Adolescent OR Female Adolescents OR Male Adolescent OR Male Adolescents OR Youth* OR Youths* OR Teens* OR Teen* OR Teenagers OR Teenager))) AND ((TS=(Sedentary Behavior) OR AB=(Sedentary behavior OR Sedentary Behaviors OR Sedentary Lifestyle OR Physical Inactivity OR Lack of Physical Activity OR Sedentary Time OR inactive OR inactivity OR Sitting) )OR (TS=(Exercise) OR AB=(Physical activity OR Exercise OR Physical Exercise OR Physical Activity OR Physical Activities OR Aerobic Exercise OR Isometric Exercise OR Acute Exercise OR Exercise Training OR Exercise Trainings OR PA OR moderate to vigorous physical activity OR moderate-to-vigorous physical activity OR leisure time activity OR leisure-time activity OR walking OR steps OR running)))

AND (TS=(Gamification) OR AB=(Gamification OR exergaming OR exer-gaming)) AND (TS=(Randomized Controlled Trial) OR AB=(Randomized Controlled Trial OR Random control test OR RCT* OR controlled clinical trial OR Randomized OR Randomly OR groups OR Trial ))

#2. ((TS=(Child) OR AB=(Children OR Child OR Preschool OR Preschool Child OR Preschool Children))OR (TS=(Adolescent) OR AB=(Adolescents OR Adolescence OR Female Adolescent OR Female Adolescents OR Male Adolescent OR Male Adolescents OR Youth* OR Youths* OR Teens* OR Teen* OR Teenagers OR Teenager))) AND ((TS=(Sedentary Behavior) OR AB=(Sedentary behavior OR Sedentary Behaviors OR Sedentary Lifestyle OR Physical Inactivity OR Lack of Physical Activity OR Sedentary Time OR inactive OR inactivity OR Sitting) )OR (TS=(Exercise) OR AB=(Physical activity OR Exercise OR Physical Exercise OR Physical Activity OR Physical Activities OR Aerobic Exercise OR Isometric Exercise OR Acute Exercise OR Exercise Training OR Exercise Trainings OR PA OR moderate to vigorous physical activity OR moderate-to-vigorous physical activity OR leisure time activity OR leisure-time activity OR walking OR steps OR running)))

AND ((TS=(game) OR AB=(games OR gaming OR gameful* OR multiplayer OR player OR playing OR Challenges OR checkpoint OR Rewards OR Combine game elements)) AND ((TS=(Telemedicine) OR AB=(telemedicine OR e-health OR m-Health OR eHealth OR mHealth)) OR (TS=(Mobile Applications) OR AB=(Mobile Applications OR applications OR application OR app OR apps OR online OR mobile OR internet)) OR (TS=(Smartphone) OR AB=(Smartphone OR phone OR smart phones OR smartphones OR phones, smart OR Smartphone OR cell phone OR iPhone OR android OR iOS) ) OR (TS=(Wearable Electronic Devices) OR AB=(Wearable Electronic Devices OR website OR digital* OR electronic* OR device)))) AND (TS=(Randomized Controlled Trial) OR AB=(Randomized Controlled Trial OR Random control test OR RCT* OR controlled clinical trial OR Randomized OR Randomly OR groups OR Trial ))

#3. (#1 OR #2)

Filters: English, from 2010-2024

**Database searched: Cochrane library (25) /Search String Embase (297)**

#1. ((((((((((((("Adolescent"[Mesh]) OR (Adolescents[Title/Abstract])) OR (Adolescence[Title/Abstract])) OR (Female Adolescent[Title/Abstract])) OR (Female Adolescents[Title/Abstract])) OR (Male Adolescent[Title/Abstract])) OR (Male Adolescents[Title/Abstract])) OR (Youth[Title/Abstract])) OR (Youths[Title/Abstract])) OR (Teens[Title/Abstract])) OR (Teen[Title/Abstract])) OR (Teenagers[Title/Abstract])) OR (Teenager[Title/Abstract])) OR ((((((("Child"[Mesh]) OR (Children[Title/Abstract])) OR (Child[Title/Abstract])) OR (Preschool[Title/Abstract])) ) OR (Preschool Child[Title/Abstract])) OR (Preschool Children[Title/Abstract]))

#2. ((((((((((((((((((("Exercise"[Mesh]) OR (Physical activity[Title/Abstract])) OR (Exercise[Title/Abstract])) OR (Physical Exercise[Title/Abstract])) OR (Physical Activity[Title/Abstract])) OR (Physical Activities[Title/Abstract])) OR (Aerobic Exercise[Title/Abstract])) OR (Isometric Exercise[Title/Abstract])) OR (Acute Exercise[Title/Abstract])) OR (Exercise Training[Title/Abstract])) OR (Exercise Trainings[Title/Abstract])) OR (PA[Title/Abstract])) OR (moderate to vigorous physical activity[Title/Abstract])) OR (moderate-to-vigorous physical activity[Title/Abstract])) OR (leisure time activity[Title/Abstract])) OR (leisure-time activity[Title/Abstract])) OR (walking[Title/Abstract])) OR (steps[Title/Abstract])) OR (running[Title/Abstract])) OR ((((((((("Sedentary Behavior"[Mesh]) OR (Sedentary behavior[Title/Abstract])) OR (Sedentary Behaviors[Title/Abstract])) OR (Sedentary Lifestyle[Title/Abstract])) OR (Physical Inactivity[Title/Abstract])) OR (Lack of Physical Activity[Title/Abstract])) OR (Sedentary Time[Title/Abstract])) OR (inactive[Title/Abstract])) OR (inactivity[Title/Abstract]))

#3. ((("Gamification"[Mesh]) OR (gamification [Title/Abstract])) OR (exergaming[Title/Abstract])) OR (exer-gaming[Title/Abstract])

#4. (((((("Randomized Controlled Trial" [Publication Type]) OR (Randomized Controlled Trial [Title/Abstract])) OR (Random control test[Title/Abstract])) OR (controlled clinical trial[Title/Abstract])) OR (Randomized[Title/Abstract])) OR (Randomly[Title/Abstract])) OR (Trial[Title/Abstract])

#5. #1 AND #2 AND #3 AND #4

#6. (((( (((((((game[Title/Abstract]) OR (games[Title/Abstract])) OR (gamified[Title/Abstract])) OR (gaming[Title/Abstract])) OR (gameful*[Title/Abstract])) OR (multiplayer[Title/Abstract])) OR (player[Title/Abstract])) OR (playing[Title/Abstract])) OR (Challenges[Title/Abstract])) OR (checkpoint[Title/Abstract])) OR (Rewards[Title/Abstract])) OR (Combine game elements[Title/Abstract])

#7. #1 AND #2 AND #4 AND #6

#8. #5 OR #7

Filters: English, from 2010-2024

**Database searched: Search String Ebsco (174)**

HREF=https://search.ebscohost.com/login.aspx?direct=true&bquery=(SU+Gamification+OR+AB+Gamification+OR+(Gamification+AND+technology)+OR+(Combine+AND+game+AND+elements))+AND+(((SU+(Randomized+AND+Controlled+AND+Trial))+OR+(AB+((Randomized+AND+Controlled+AND+Trial)+OR+(Random+AND+control+AND+test)+OR+RCT*+OR+(controlled+AND+clinical+AND+trial)+OR+Randomized+OR+Randomly+OR+groups+OR+Trial)))+AND+(((SU+(Sedentary+AND+Behavior))+OR+(AB+((Sedentary+AND+behavior)+OR+(Sedentary+AND+Behaviors)+OR+(Sedentary+AND+Lifestyle)+OR+(Physical+AND+Inactivity)+OR+(Lack+AND+%26quot%3bof%26quot%3b+AND+Physical+AND+Activity)+OR+(Sedentary+AND+Time)+OR+inactive+OR+inactivity)))+OR+((SU+Exercise)+OR+(AB+((Physical+AND+activity)+OR+Exercise+OR+(Physical+AND+Exercise)+OR+(Physical+AND+Activity)+OR+(Physical+AND+Activities)+OR+(Aerobic+AND+Exercise)+OR+(Isometric+AND+Exercise)+OR+(Acute+AND+Exercise)+OR+(Exercise+AND+Training)+OR+(Exercise+AND+Trainings)+OR+PA+OR+(moderate+AND+%26quot%3bto%26quot%3b+AND+vigorous+AND+physical+AND+activity)+OR+(moderate-to-vigorous+AND+physical+AND+activity)+OR+(leisure+AND+time+AND+activity)+OR+(leisure-time+AND+activity)+OR+walking+OR+steps+OR+running))))+AND+(((SU+Child)+OR+(AB+(Children+OR+Child+OR+Preschool+OR+(Preschool+AND+Child)+OR+(Preschool+AND+Children))))+OR+((SU+Adolescent)+OR+(AB+(Adolescents+OR+Adolescence+OR+(Female+AND+Adolescent)+OR+(Female+AND+Adolescents)+OR+(Male+AND+Adolescent)+OR+(Male+AND+Adolescents)+OR+Youth+OR+Youths+OR+Teens+OR+Teen+OR+Teenagers+OR+Teenager))))+AND+((SU+Gamification)+OR+(AB+(Gamification+OR+(Gamification+AND+technology)+OR+(Combine+AND+game+AND+elements)))))&lang=zh-cn&type=1&searchMode=And&site=eds-live&ssl=y>(SU Gamification OR AB Gamification OR Gamification technology OR Combine game elements) AND (S18 AND S19 AND S20 AND S22)</A>
