# Supplementary material for: Effectiveness of Gamification Interventions to Improve Physical Activity and Sedentary Behavior in Children and Adolescents: Systematic Review and Meta-Analysis
Source: JMIR Serious Games. 2025 Sep 18;13:e68151. doi: 10.2196/68151 (PMC12445784; doi:10.2196/68151)
Supplement: Multimedia Appendix 2 [file games-v13-e68151-s002.pdf]

Multimedia Appendix 2: Sensitivity analyses results on light physical activity.

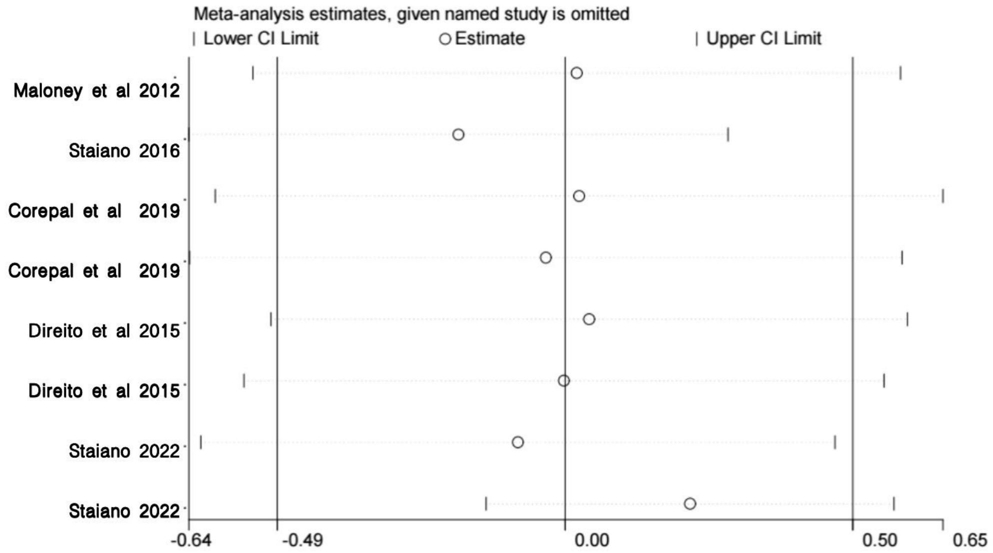

1. Corepal R, Best P, O'Neill R, et al. A feasibility study of "The StepSmart Challenge" to promote physical activity in adolescents. Pilot Feasibility Stud. 2019;5:132. [doi:10.1186/s40814-019-0523-5] [Medline: 31832227]
2. Direito A, Jiang Y, Whittaker R, Maddison R. Apps for IMproving FITness and increasing physical activity among young people: the AIMFIT pragmatic randomized controlled trial. J Med Internet Res. Aug 27, 2015;17(8):e210. [doi: 10.2196/jmir.4568] [Medline: 26316499]
3. Maloney AE, Threlkeld KA, Cook WL. Comparative effectiveness of a 12-week physical activity intervention for overweight and obese youth: exergaming with "Dance Dance Revolution". Games Health J. Apr 2012;1(2):96-103. [doi: 10.1089/g4h.2011.0009] [Medline: 26193183]
4. Staiano AE, Beyl RA, Hsia DS, Katzmarzyk PT, Newton RL Jr. Twelve weeks of dance exergaming in overweight and obese adolescent girls: transfer effects on physical activity, screen time, and self-efficacy. J Sport Health Sci. Mar 2017;6(1):4-10. [doi: 10.1016/j.jshs.2016.11.005] [Medline: 28491483]
5. Staiano AE, Newton RL, Beyl RA, et al. mHealth intervention for motor skills: a randomized controlled trial. Pediatrics. May 1, 2022;149(5):e2021053362. [doi: 10.1542/peds.2021-053362] [Medline: 35415743]
