# Supplementary material for: Effectiveness of Gamification Interventions to Improve Physical Activity and Sedentary Behavior in Children and Adolescents: Systematic Review and Meta-Analysis
Source: JMIR Serious Games. 2025 Sep 18;13:e68151. doi: 10.2196/68151 (PMC12445784; doi:10.2196/68151)
Supplement: Multimedia Appendix 3 [file games-v13-e68151-s003.pdf]

Multimedia Appendix 3 Sensitivity analyses result on sedentary behavior.

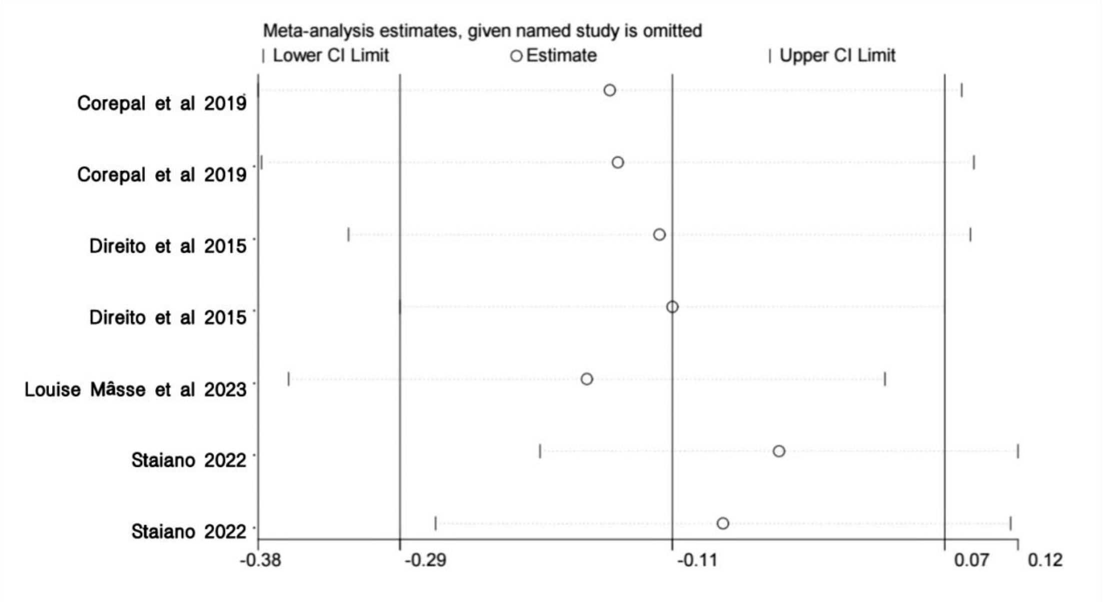

1. Corepal R, Best P, O'Neill R, et al. A feasibility study of "The StepSmart Challenge" to promote physical activity in adolescents. Pilot Feasibility Stud. 2019;5:132. [doi: 10.1186/s40814-019-0523-5] [Medline: 31832227]
2. Direito A, Jiang Y, Whittaker R, Maddison R. Apps for IMproving FITness and increasing physical activity among young people: the AIMFIT pragmatic randomized controlled trial. J Med Internet Res. Aug 27, 2015;17(8):e210. [doi: 10.2196/jmir.4568] [Medline: 26316499]
3. Staiano AE, Newton RL, Beyl RA, et al. mHealth intervention for motor skills: a randomized controlled trial. Pediatrics. May 1, 2022;149(5):e2021053362. [doi: 10.1542/peds.2021-053362] [Medline: 35415743]
4. Tugault-Lafleur CN, De-Jongh González O, Macdonald J, et al. Efficacy of the Aim2Be intervention in changing lifestyle behaviors among adolescents with overweight and obesity: randomized controlled trial. J Med Internet Res. Apr 25, 2023;25:e38545. [doi: 10.2196/38545] [Medline: 37097726]
