# Supplementary material for: Effectiveness of Gamification Interventions to Improve Physical Activity and Sedentary Behavior in Children and Adolescents: Systematic Review and Meta-Analysis
Source: JMIR Serious Games. 2025 Sep 18;13:e68151. doi: 10.2196/68151 (PMC12445784; doi:10.2196/68151)
Supplement: Multimedia Appendix 4 [file games-v13-e68151-s004.pdf]

Multimedia Appendix 4 Sensitivity analyses result on moderate-to-vigorous physical activity.

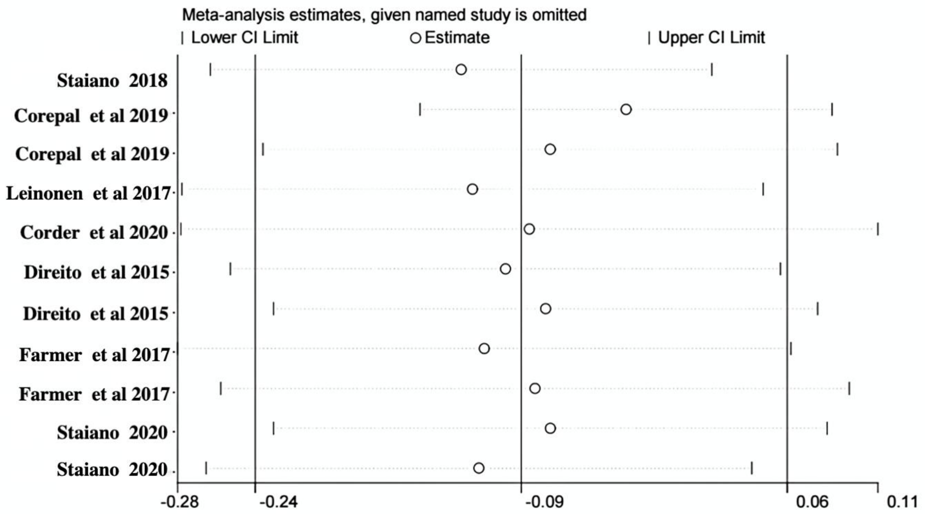

1. Corder K, Sharp SJ, Jong ST, et al. Effectiveness and cost-effectiveness of the GoActive intervention to increase physical activity among UK adolescents: a cluster randomised controlled trial. PLoS Med. Jul 2020;17(7):e1003210. [doi: 10.1371/journal.pmed.1003210] [Medline: 32701954]
2. Corepal R, Best P, O'Neill R, et al. A feasibility study of "The StepSmart Challenge" to promote physical activity in adolescents. Pilot Feasibility Stud. 2019;5:132. [doi: 10.1186/s40814-019-0523-5] [Medline: 31832227]
3. Direito A, Jiang Y, Whittaker R, Maddison R. Apps for IMproving FITness and increasing physical activity among young people: the AIMFIT pragmatic randomized controlled trial. J Med Internet Res. Aug 27, 2015;17(8):e210. [doi: 10.2196/jmir.4568] [Medline: 26316499]
4. Farmer VL, Williams SM, Mann JI, Schofield G, McPhee JC, Taylor RW. The effect of increasing risk and challenge in the school playground on physical activity and weight in children: a cluster randomised controlled trial (PLAY). Int J Obes (Lond). May 2017;41(5):793-800. [doi: 10.1038/ijo.2017.41] [Medline: 28186099]
5. Leinonen AM, Pyky R, Ahola R, et al. Feasibility of gamified mobile service aimed at physical activation in young men. JMIR mHealth uHealth. 2017;5(10). [doi: 10.2196/mhealth.6675] [Medline: 29017991]
6. Staiano AE, Beyl RA, Guan W, Hendrick CA, Hsia DS, Newton RL Jr. Home-based exergaming among children with overweight and obesity: a randomized clinical trial. Pediatr Obes. Nov 2018;13(11):724-733. [doi: 10.1111/ijpo.12438] [Medline: 30027607]
7. Staiano AE, Newton RL, Beyl RA, et al. mHealth intervention for motor skills: a randomized controlled trial. Pediatrics. May 1, 2022;149(5):e2021053362. [doi: 10.1542/peds.2021-053362] [Medline: 35415743]
