# Supplementary material for: Effectiveness of Gamification Interventions to Improve Physical Activity and Sedentary Behavior in Children and Adolescents: Systematic Review and Meta-Analysis
Source: JMIR Serious Games. 2025 Sep 18;13:e68151. doi: 10.2196/68151 (PMC12445784; doi:10.2196/68151)
Supplement: Multimedia Appendix 5 [file games-v13-e68151-s005.doc]

Summary of subgroup analysis results of on moderate to vigorous physical activity

| Potential modifiers | Studies, n | Tests of heterogeneity | | | Effect size（95%CI） | | |
| --- | --- | --- | --- | --- | --- | --- | --- |
|  |  | Z | P | I^2^(%) |  |  |  |
| **MVPA** |  |  |  |  |  |  |  |
| Pooled effect size | 11 | 2.07 | 0.04 | 68 | 0.15 | 0.01 | 0.29 |
| **Age(years)** |  |  |  |  |  |  |  |
| 1-11 | 6 | 0.29 | 0.77 | 63 | -0.03 | -0.25 | 0.19 |
| 12-18 | 5 | 1.69 | 0.09 | 76 | 0.20 | 0.03 | 0.43 |
| **Theoretical paradigm** |  |  |  |  |  |  |  |
| SCT | 3 | 0.65 | 0.52 | 72.6 | -0.19 | -0.76 | 0.39 |
| SDT | 3 | 2.02 | 0.04 | 91 | 0.39 | 0.01 | 0.77 |
| BCT and other theories | 5 | 0.2 | 0.84 | 25 | 0.01 | -0.11 | 0.13 |
| **gamification element** |  |  |  |  |  |  |  |
| Social and interactive | 5 | 0.33 | 0.74 | 58 | -0.07 | -0.46 | 0.33 |
| Rewards, feedback, etc. | 6 | 2.02 | 0.04 | 86 | 0.19 | 0.01 | 0.37 |
| **duration** |  |  |  |  |  |  |  |
| ≦12weeks | 7 | 0.21 | 0.83 | 76.9 | 0.02 | -0.15 | 0.19 |
| >12weeks | 4 | 2.32 | 0.02 | 1.1 | 0.14 | 0.02 | 0.26 |
| **test environment** |  |  |  |  |  |  |  |
| Family (community) environment | 5 | 0.58 | 0.55 | 48 | -0.08 | -0.35 | 0.20 |
| School environment | 6 | 2.07 | 0.04 | 79 | 0.18 | 0.01 | 0.36 |
| **measuring tools** |  |  |  |  |  |  |  |
| Hip (or belt fixation) | 7 | 0.04 | 0.97 | 52 | 0.00 | -0.20 | 0.19 |
| wrist | 4 | 1.56 | 0.12 | 84.6 | 0.21 | -0.06 | 0.48 |
| **number** |  |  |  |  |  |  |  |
| ≦100 | 6 | 1.7 | 0.09 | 78 | 0.14 | -0.02 | 0.29 |
| >100 | 5 | 0.33 | 0.74 | 58 | -0.07 | -0.46 | 0.33 |
