# Supplementary figures and images for: Effectiveness of Gamification Interventions to Improve Physical Activity and Sedentary Behavior in Children and Adolescents: Systematic Review and Meta-Analysis
Source: JMIR Serious Games. 2025 Sep 18;13:e68151. doi: 10.2196/68151 (PMC12445784; doi:10.2196/68151)

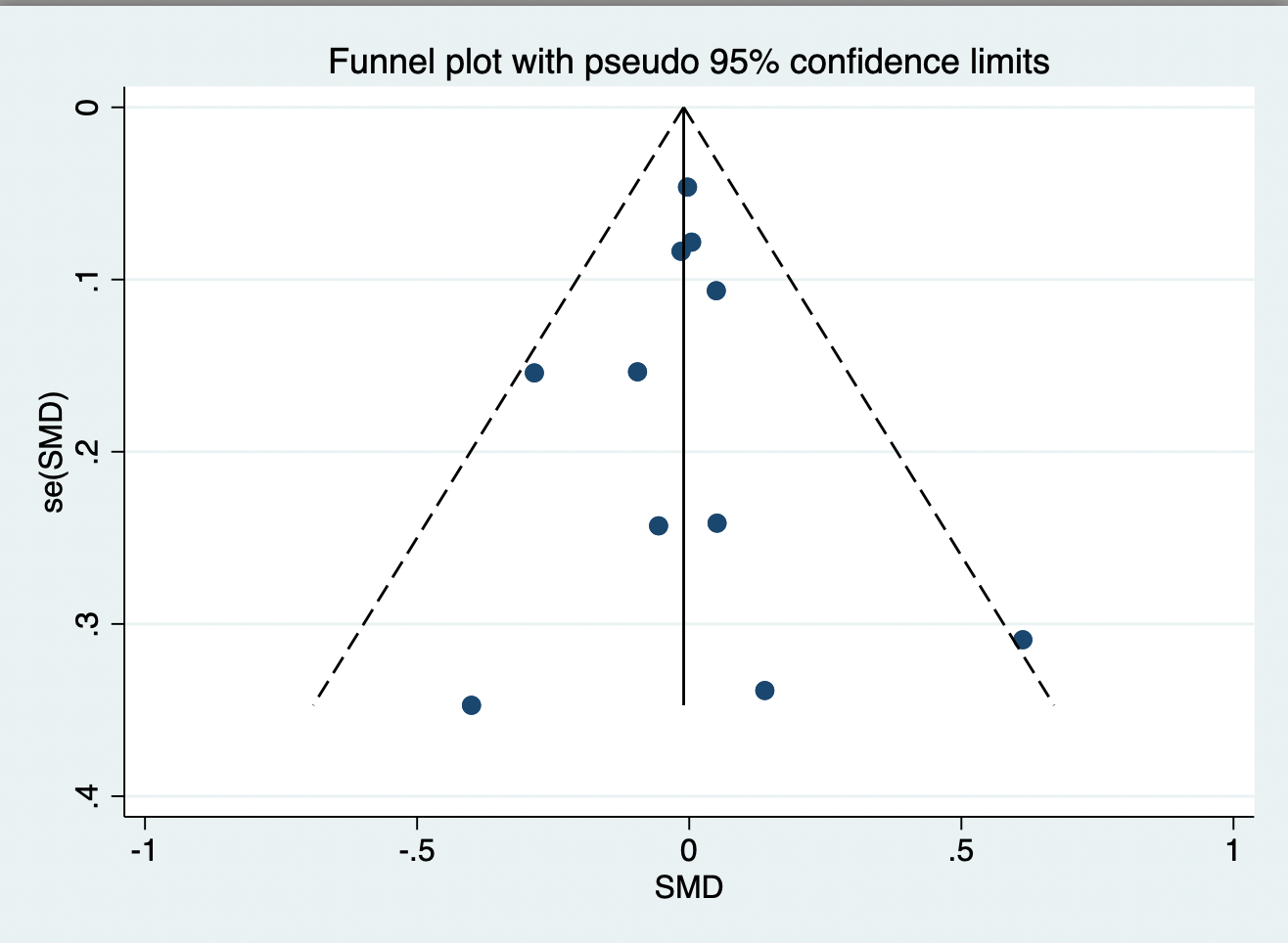

Supplement: Multimedia Appendix 6 [file games-v13-e68151-s006.png]

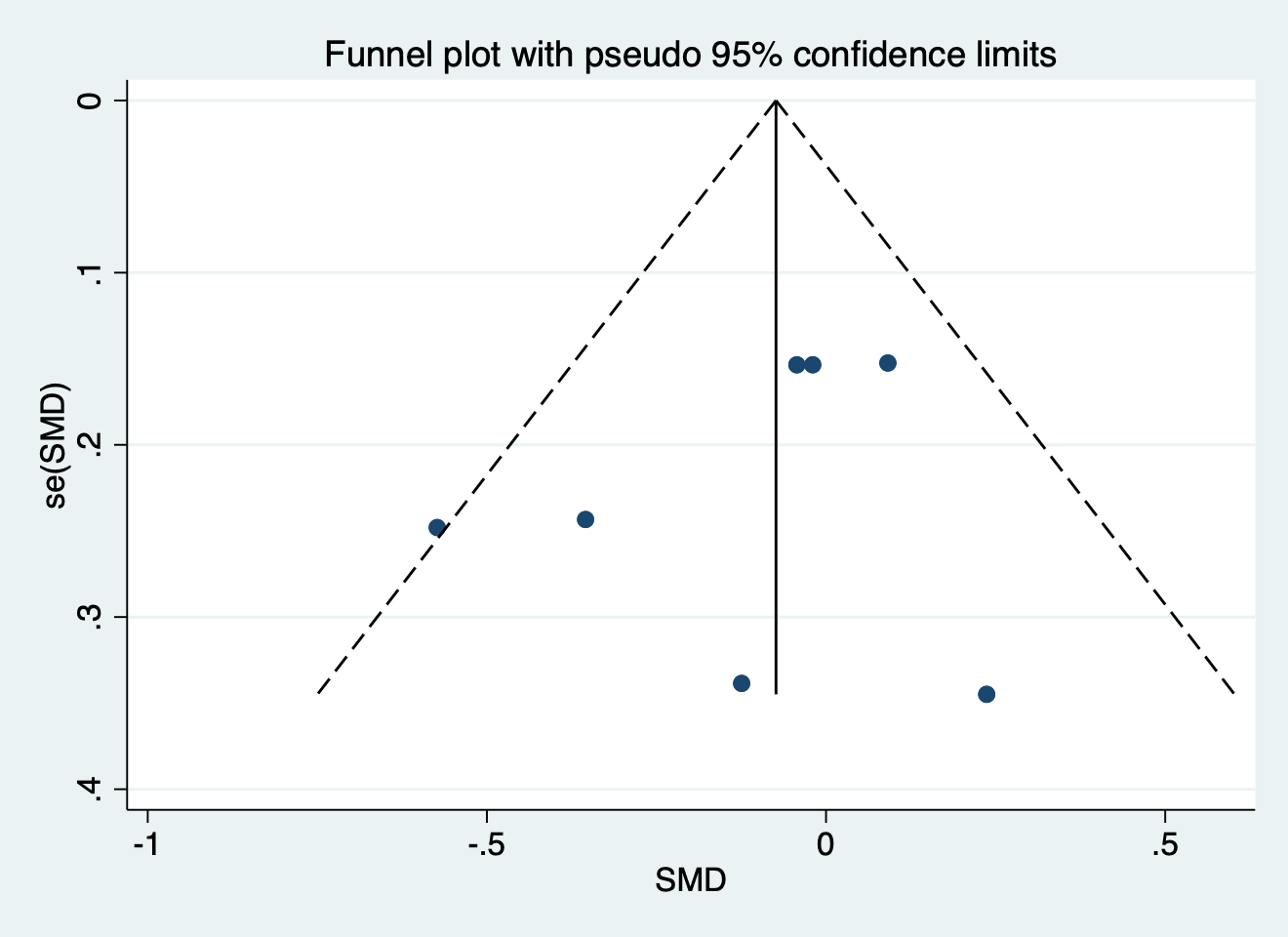

Supplement: Multimedia Appendix 7 [file games-v13-e68151-s007.png]

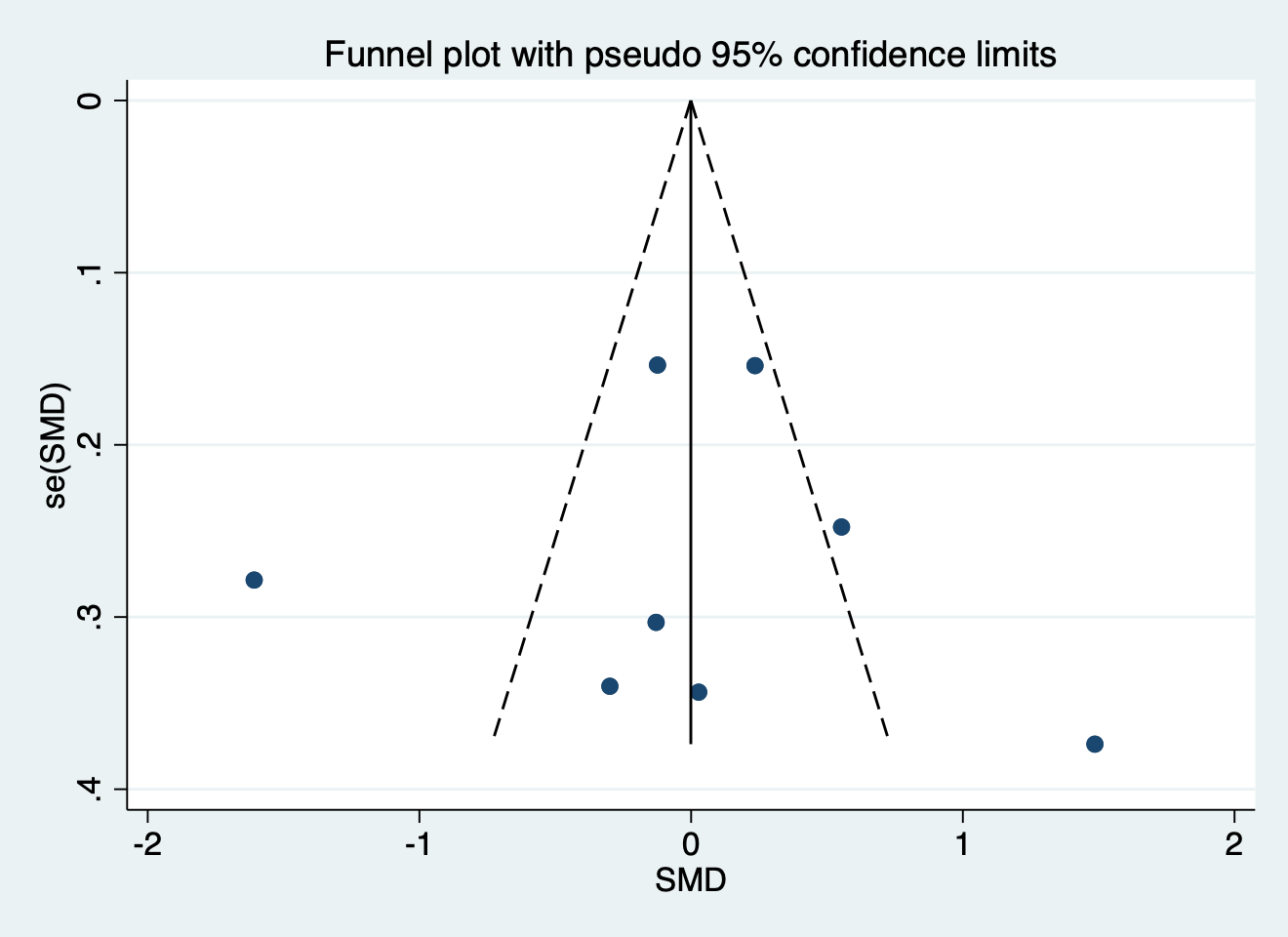

Supplement: Multimedia Appendix 8 [file games-v13-e68151-s008.png]

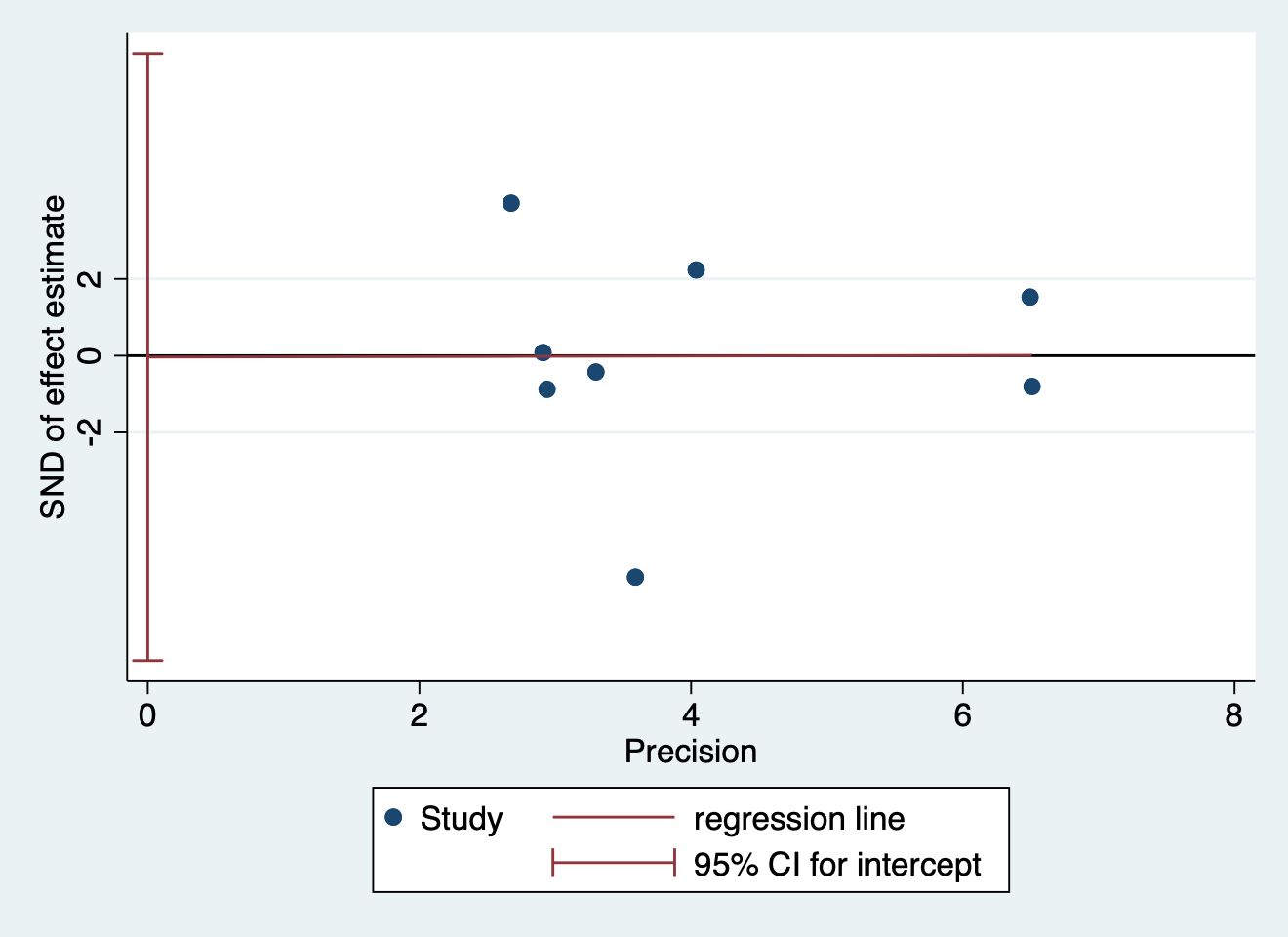

Supplement: Multimedia Appendix 9 [file games-v13-e68151-s009.png]

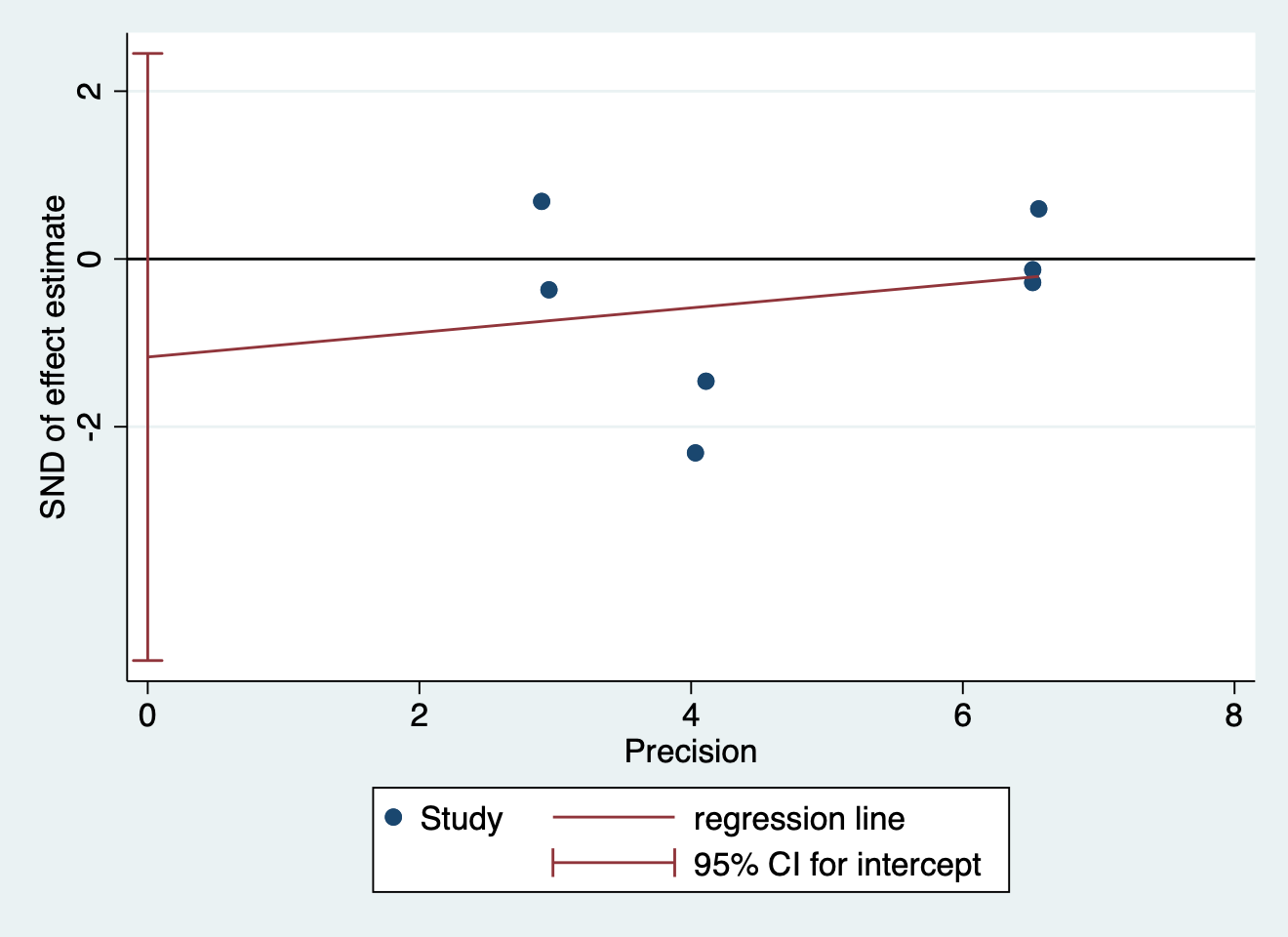

Supplement: Multimedia Appendix 10 [file games-v13-e68151-s010.png]

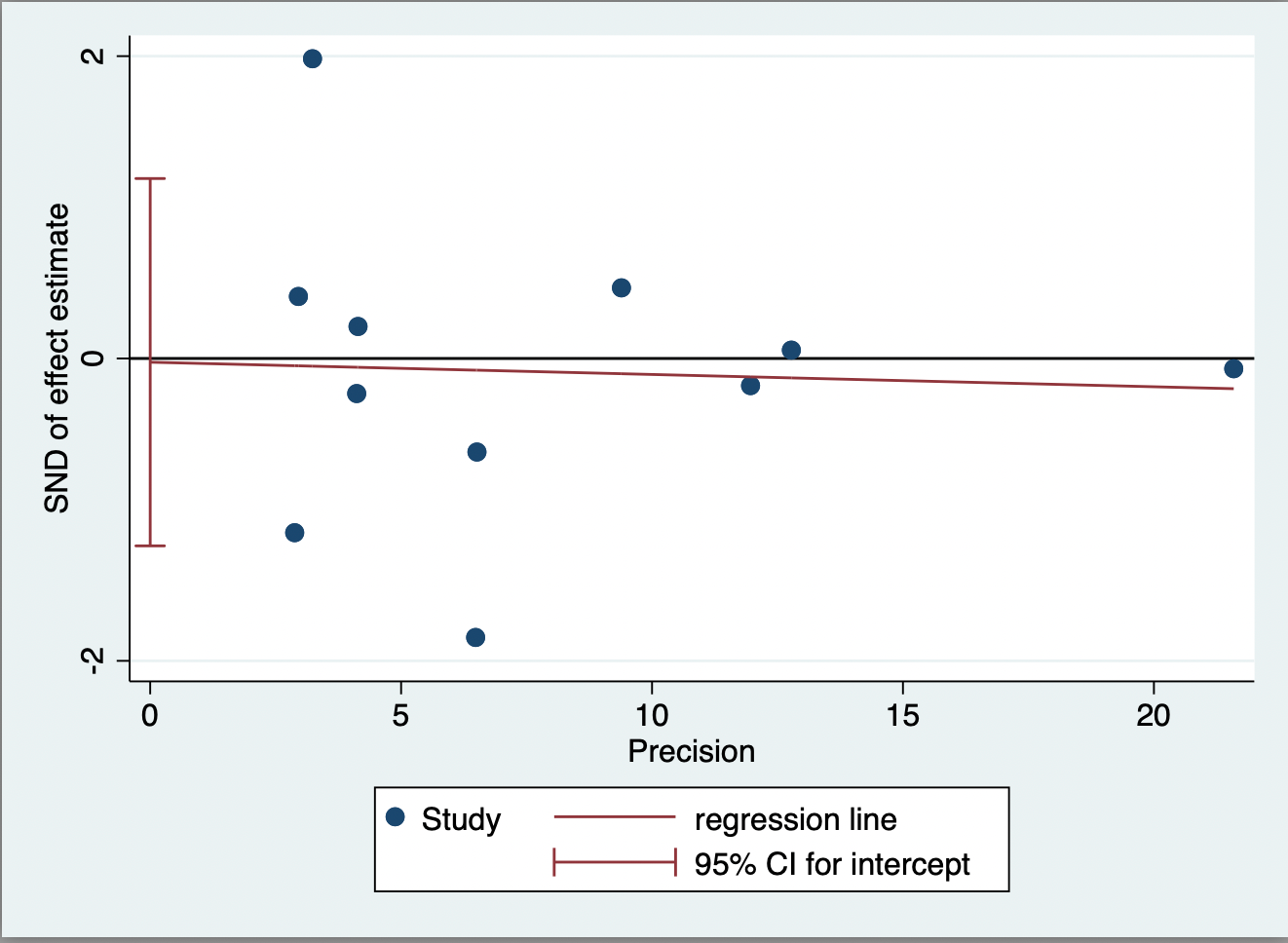

Supplement: Multimedia Appendix 11 [file games-v13-e68151-s011.png]
